# Comparison of single-channel EEG, actigraphy, and sleep diary in cognitively normal and mildly impaired older adults

**DOI:** 10.1093/sleepadvances/zpaa006

**Published:** 2020-10-24

**Authors:** Chris A Chou, Cristina D Toedebusch, Tiara Redrick, David Freund, Jennifer S McLeland, John C Morris, David M Holtzman, Brendan P Lucey

**Affiliations:** 1 Department of Neurology, Washington University School of Medicine, St Louis, MO; 2 Hope Center for Neurological Disorders, Washington University School of Medicine, St Louis, MO; 3 Knight Alzheimer Disease Research Center, Washington University School of Medicine, St Louis, MO

**Keywords:** actigraphy, dementia, home testing, neurodegenerative disorders

## Abstract

**Study Objectives:**

Multiple methods for monitoring sleep-wake activity have identified sleep disturbances as risk factors for Alzheimer disease (AD). In order to identify the level of agreement between different methods, we compared sleep parameters derived from single-channel EEG (scEEG), actigraphy, and sleep diaries in cognitively normal and mildly impaired older adults.

**Methods:**

Two hundred ninety-three participants were monitored at home for up to six nights with scEEG, actigraphy, and sleep diaries. Total sleep time (TST), sleep efficiency (SE), sleep onset latency (SOL), and wake after sleep onset (WASO) were calculated using each of these methods. In 109 of the 293 participants, the ratio of cerebrospinal fluid concentrations of phosphorylated tau (p-tau) and amyloid-β-42 (Aβ42) was used as a biomarker for AD pathology.

**Results:**

Agreement was highest for TST across instruments, especially in cognitively normal older adults. Overall, scEEG and actigraphy appeared to have greater agreement for multiple sleep parameters than for scEEG and diary or actigraphy and diary. Levels of agreement between scEEG and actigraphy overall decreased in mildly impaired participants and those with biomarker evidence of AD pathology, especially for measurements of TST.

**Conclusions:**

Caution should be exercised when comparing scEEG and actigraphy in individuals with mild cognitive impairment or with AD pathology. Sleep diaries may capture different aspects of sleep compared to scEEG and actigraphy. Additional studies comparing different methods of measuring sleep-wake activity in older adults are necessary to allow for comparison between studies using different methods.

**Statement of Significance**

Sleep-wake activity may be monitored using a number of different instruments. In this study, sleep-wake activity was monitored in 293 cognitively normal and mildly impaired older adults for up to 6 nights using three different methods simultaneously: (1) single-channel EEG device worn on the forehead; (2) actigraphy; (3) sleep diary. Participants also underwent standardized cognitive assessments and a subset of 109 participants had CSF Alzheimer disease (AD) biomarkers measured. This is one of the largest cohorts comparing subjective and objective sleep monitoring methods over multiple nights, and no previous study has participants as well-characterized for both cognitive function and CSF AD biomarkers. Understanding how different sleep instruments measure sleep-wake activity in older adults with and without evidence of AD will help the sleep field compare studies using different methods.

## Introduction

Alzheimer disease (AD) is a progressive neurodegenerative disorder characterized by the accumulation of amyloid plaques in the brain, intracellular tangles of tau, synaptic and neuronal loss, cognitive dysfunction, and eventual dementia [1, 2]. Sleep disturbance is common in AD with reports that around 40% of patients with AD suffer from sleep problems [3]. Changes in sleep comprise an important component of disability and lifestyle disruption for the elderly and their caretakers, increasing the risk of early institutionalization [4]. Given the rising prevalence of AD, there has been increasing interest in identifying additional early markers of AD pathology. Sleep disturbance is hypothesized to be both an early marker of preclinical AD as well as a risk factor for developing AD pathology itself [5, 6]. Polysomnographic, actigraphic, and subjective evidence have all demonstrated sleep disturbance associated with early symptomatic AD (this term encompasses individuals labeled with mild cognitive impairment (MCI) due to AD and very mild AD dementia or with preclinical AD) [7–13]. Changes in sleep duration with aging have been associated not only with cognitive impairment, but also with premature mortality [14], cardiovascular disease [15], and physical frailty [16, 17]. Further investigation is needed, but the challenge remains to identify the optimal diagnostic tools for assessing sleep in the elderly on a large scale while balancing ease of use, accuracy of measurement, and cost-effectiveness.

Attended polysomnography (PSG) in a sleep laboratory is the gold standard for assessing sleep [18]. However, PSG can be costly as well as inconvenient for older adults due to the need to travel outside the home. Furthermore, sleeping in a new, unfamiliar environment can be disruptive to sleep and serves as the basis for the “first night” effect seen with PSG [19]. Other objective instruments that allow testing in the naturalistic home environment such as single-channel EEG (scEEG) and wrist-worn actigraphy have been investigated as alternative and/or supplementary methods of measuring sleep accurately and accessibly. scEEG is a noninvasive forehead-worn device that allows for measuring sleep stages with one frontal EEG channel. Previous work has demonstrated that scEEG has a high level of agreement with PSG for sleep staging and measuring sleep parameters, with the caveat that scEEG appears to have more difficulty determining transitions between sleep-wake stages, and agreement with PSG worsens with increased sleep fragmentation [20–22]. Wrist-worn actigraphy relies on the user’s movement activity to estimate sleep and wakefulness. It has been well-described as a valid method of measuring sleep in comparison to PSG [23, 24], though it is not validated for staging sleep and may be prone to overestimation of sleep due to difficulties in distinguishing motionless wake from sleep [25]. In one study of healthy young adults from Japan [26], scEEG had greater correlation and agreement with PSG than actigraphy for certain sleep parameters such as wake after sleep onset (WASO). However, studies performing similar comparisons in older adults are needed.

Subjective measures of sleep are important in assessing sleep. However, studies comparing objective sleep (including as assessed by PSG) to subjective sleep diaries or questionnaires in both younger adults and cognitively normal older adults have found limited association between instruments, especially in regards to perceived sleep quality [27–35]. For sleep measurements that were found to correlate, agreement assessed by Bland-Altman plots was generally poor [27, 34, 36, 37]. Little association between subjective and objective sleep has been demonstrated in patients with early symptomatic AD [38–40]. Further, many of the studies in older adults comparing one objective to one subjective instrument have been limited by sample sizes of less than 100 participants. Our study is a cross-sectional analysis of a cohort of nearly 300 cognitive normal or very mildly symptomatic participants ≥60 years old that aims to compare the level of agreement for three at-home sleep-wake monitoring instruments.

## Methods

### Study design

Participants were enrolled in an ongoing longitudinal observational study of aging and AD at the Knight Alzheimer Disease Research Center at Washington University in St. Louis, MO. Sleep data included in this analysis was collected from 2013 to 2019. Participants were ≥60 years old and underwent a clinical assessment that included the Clinical Dementia Rating (CDR) [41] and Mini Mental State Examination (MMSE) [42]. Participants were included if they scored CDR 0 (cognitively normal) or CDR 0.5 (very mild AD dementia).

### Sleep monitoring

The protocols for sleep monitoring in this study have been previously described [7, 43, 44]. Sleep was assessed over six nights at home using three instruments simultaneously: scEEG (Sleep Profiler, Advanced Brain Monitoring, Carlsbad, CA), actigraphy (Actiwatch2, Philips Respironics, Andover, MA), and sleep diaries. Actigraphy and sleep diaries were used to confirm that the scEEG recorded the entire sleep period. The scEEG device was worn on the forehead and recorded at 256 samples per second from three frontal sensors placed at approximately AF7, AF8, and Fpz. Only the AF7-AF8 channel was used for scoring. The scEEG hardware applies a 0.1 Hz low-frequency filter and a 67 Hz high-frequency filter. The scEEG studies were scored by registered polysomnographic technologists using modified American Academy of Sleep Medicine (AASM) criteria [20]. Actigraphy data were processed using Actiware (Phillips Respironics) with the wake threshold at the “low” setting of 20, which has been shown to correspond best with PSG [23]. Total sleep time (TST), sleep efficiency (SE), sleep onset latency (SOL), and WASO were calculated using the data from each instrument.

Participants were instructed to complete the sleep diary every morning upon awakening and documented bedtime, wake-up time, TST, how long it took to fall asleep (SOL), number of nighttime awakenings, and occurrence of unusual events that may impact sleep. Since SE and WASO were not recorded by the participants, we calculated diary SE by dividing reported TST by reported time in bed (TIB; time between bedtime and wake-up time) and diary WASO by subtracting TST and SOL from TIB. A minority of participants had SE and WASO parameters calculated using the sleep diary that led to SE >100% (17 participants, 76% CDR 0) and WASO <0 minutes (83 participants, 80% CDR 0). These data were kept in the analysis since they were actual participant responses on the questionnaire with the exception of one participant whose diary-calculated SE for a single night was an extreme outlier.

Additionally, participants underwent a one-night home sleep apnea test (HSAT) (Alice PDx, Philips Respironics) to assess for sleep-disordered breathing or periodic limb movements during sleep. Those already using positive airway pressure (PAP) therapy were instructed to continue treatment during monitoring with the HSAT. All recordings were reviewed by a board-certified sleep physician.

### Cerebrospinal fluid biomarkers

Cerebrospinal fluid (CSF) was collected under a standardized protocol [45] within one year of sleep monitoring [44]. Participants underwent a fasting morning lumbar puncture and CSF was collected by gravity drip and stored in polypropylene tubes at -80°C until analysis. CSF total tau, phosphorylated tau-181 (p-tau), and amyloid-β-peptide 42 (Aβ42) were measured using an automated electrochemiluminescence immunoassay (Elecsys immunoassay on the cobas e 601 analyzer, Roche, Indianapolis, IN, USA). The ratio of CSF p-tau to Aβ42 (p-tau/Aβ42) was calculated as a measure of AD pathology using previously defined cutoffs [45]: a higher CSF p-tau/Aβ42 ratio signifies more AD pathology [46].

### Data analysis

We included participants who had data available for at least 2 nights with all of the three instruments and calculated the average of each sleep parameter for use in our analysis. Paired sample *t*-tests were used to calculate mean differences and 95% confidence intervals (CIs). To assess acceptable clinical agreement between instruments, we used methods from a recently published systematic review and meta-analysis on the use of actigraphy from an AASM-commissioned task force [47] that defined clinically significant thresholds of agreement based on clinical expertise of the task force. These guidelines establish minimum thresholds of agreement between sleep instruments for diagnostic purposes (i.e. measurement differences between devices do not change a diagnosis); this level of precision may not be adequate for research purposes. The clinically significant thresholds of agreement were determined between objective instruments (actigraphy vs. PSG) using 95% CIs of the mean difference, and objective vs. subjective instruments (actigraphy vs. sleep logs) using the mean difference. Since there were no published criteria comparing scEEG to actigraphy or scEEG to diary, we applied the standard for PSG to scEEG. Following this criteria, the maximum allowable 95% CIs of the mean difference between objective instruments were 40 minutes for TST, 5% for SE, 30 minutes for SOL, and 30 minutes for WASO. A 95% CI of the mean difference within these limits is considered narrow enough that the two objective instruments do not have clinically significant differences and can be used interchangeably to provide consistent, objective sleep measurements. A 95% CI above these limits is considered a clinically significant difference. The allowable mean differences between objective and subjective instruments were 20 minutes for TST, 2.5% for SE, 15 minutes for SOL, and 15 minutes for WASO. A difference above these thresholds is considered a clinically significant difference such that diaries cannot be considered as providing similar data as the objective method and it is recommended to use an objective instrument to provide the measurement.

Intraclass correlation coefficients (ICCs) were calculated for each sleep parameter and instrument pair (scEEG vs. actigraphy, scEEG vs. diary, actigraphy vs. diary), and are sensitive to differences in the means of the observations and are a measure of interobserver agreement [48]. ICC values of <0.5, 0.5–0.75, 0.75–0.9, and >0.9 indicate poor, moderate, good, and excellent reliability, respectively [49]. One-way analysis of variance (ANOVA) were used to compare group differences (e.g. CDR 0 vs. CDR 0.5) in sleep parameters (e.g. TST, SE, etc.) measured by the same instrument (e.g. scEEG). Finally, Bland-Altman plots were generated for all sleep parameters and instrument comparisons [50].

Bivariate Pearson correlation coefficients were calculated to assess linear relationships between instruments and these results are available in the Supplementary Materials (Supplementary Table 3, Supplementary Figures 1–6).

All statistical analyses were performed using SPSS version 26 (IBM Corporation, Armonk, NY). Bland-Altman plots, scatterplots, and linear regression best-fit lines were created on GraphPad version 8.4.2 for Mac (GraphPad Software, San Diego, CA). Statistical significance was set at *p* < 0.05.

## Results

### Demographics

Participant characteristics are shown in Table 1. A total of 293 participants had data available for all three instruments. The mean age was 72.8 years (standard deviation [SD] = 10.1); 136 (46.4%) participants were male. Overall, the average apnea-hypopnea index was mild (mean 8.98 events/hour, SD = 9.39) as was the average periodic limb movement index (mean 24.4 events/hour, SD = 26.4). 235 participants had scores available for CDR and MMSE. 81.7% of participants were CDR 0 and 90.2% of participants had a MMSE ≥27, often considered to represent normal cognition [51]. CSF p-tau/Aβ42 data was available for 109 participants. 58.7% of participants had a low CSF p-tau/Aβ42, indicating no evidence of AD brain pathology [45].

**Table 1.** Participant characteristics

Variable	Mean (σ) or *n* (%)	Range
Age (years, *N* = 293)	72.8 (10.1)	61–91
Sex (*N* = 293)	---	---
Male	136 (46.4%)	
Female	157 (53.6%)	
AHI (events/hour, *N* = 287)	8.98 (9.39)	0–48.9
Negative (AHI <5)	131 (45.6%)	
Mild (AHI 5–15)	103 (35.9%)	
Moderate (AHI 15–30)	42 (14.6%)	
Severe (AHI ≥30)	11 (3.83%)	
Lowest O_2_ saturation (%, *N* = 287)	82.2 (6.85)	53–93%
PLMI (events/hour, *N* = 284)	24.4 (26.4)	0–121.8
Negative (PLMI <15)	150 (52.8%)	
Low (PLMI 15–45)	68 (23.9%)	
High (PLMI ≥45)	66 (23.2%)	
CDR (*N* = 235)	---	---
CDR 0	192 (81.7%)	
CDR 0.5	43 (18.3%)	
MMSE (*N* = 235)	28.8 (1.64)	21–30
Normal cognition (MMSE ≥27)	212 (90.2%)	
Mild cognitive impairment (MMSE <27)	23 (9.8%)	
p-tau/Aβ42 (*N* = 109)	0.0264 (0.0264)	0.00733–0.166
Low p-tau/Aβ42 (≤0.0198)	64 (58.7%)	
High p-tau/Aβ42 (>0.0198)	45 (41.3%)	

*N* = number of participants with available data for specified variable. AHI = apnea/hypopnea index. PLMI = periodic limb movement index. CDR = Clinical Dementia Rating. MMSE = Mini Mental State Examination. p-tau/Aβ42 = ratio of cerebrospinal fluid phosphorylated tau to amyloid-β-42 peptide.

### Sleep parameters

Mean sleep parameters are shown for the average of all available nights in Table 2. The average sleep parameters for individual nights are shown in Supplementary Table 1, the number of participants with 2, 3, 4, 5, or 6 nights of sleep monitoring are shown in Supplementary Table 2, and the number of participants with data available and correlations for individual nights are shown in Supplementary Table 3. Our study population demonstrated average SE, SOL, and WASO at the borderline or sufficient to meet criteria for insomnia [52–54]. SE was <90% for scEEG (78.7%), actigraphy (80.9%), and diary (89.3%). SOL measurements clustered around 20 minutes for each night (mean SOL for scEEG: 17.6 minutes; actigraphy: 21.9 minutes; sleep diary: 21.3 minutes). WASO measurements were close to 1 hour for each night (mean WASO for scEEG: 71.6 minutes; actigraphy: 56.6 minutes; sleep diary: 41.6 minutes).

**Table 2.** Average sleep parameters for each instrument

	Mean (SD)
scEEG	
TST (minutes)	373.7 (59.1)
SE (%)	78.7 (9.3)
SOL (minutes)	17.6 (12.4)
WASO (minutes)	80.0 (46.5)
Actigraphy	
TST (minutes)	390.6 (58.0)
SE (%)	80.9 (8.4)
SOL (minutes)	21.9 (21.1)
WASO (minutes)	56.6 (23.1)
Diary	
TST (minutes)	435 (63.3)
SE (%)	89.3 (10.5)
SOL (minutes)*	24.7 (21.3)
WASO (minutes)	41.6 (83.0)

*There were 289 participants with Diary SOL and WASO; other sleep parameters were available for all 293 participants.

### Agreement between sleep instruments

To assess agreement between instruments, we compared the mean differences and 95% CIs between each instrument according to previously published criteria [47]. The ranges of the 95% CIs between scEEG and actigraphy were within the cutoffs for TST, SE, SOL, and WASO to not be considered clinically significant differences (Table 3). However, when comparing the scEEG or actigraphy to the diary, mean differences for TST and SE were clinically significant (Table 3). On the other hand, the mean differences for SOL and WASO between scEEG or actigraphy and the diary were not clinically significant. Although several of the comparisons were deemed clinically significant by this criteria, we note that there was a statistical difference for all comparisons except for SOL measured by actigraphy and diary (*p* = 0.063, Table 3).

**Table 3.** Mean differences and paired *t*-tests

		95% CI of mean difference			
	Mean difference	Lower	Upper	95% CI range	*P*
scEEG—actigraphy					
TST (minutes)	−16.94	−23.31	−10.57	**12.74**	<0.0001
SE (%)	−2.14	−3.34	−0.93	**2.41**	0.001
SOL (minutes)	−4.28	−6.67	−1.88	**4.79**	0.001
WASO (minutes)	23.42	18.05	28.79	**10.74**	<0.0001
scEEG—diary					
TST (minutes)	−*61.84*	−69.12	−54.56	14.56	<0.0001
SE (%)	−*10.55*	−12.03	−9.07	2.96	<0.0001
SOL (minutes)	−**7.21**	−9.53	−4.89	4.64	<0.0001
WASO (minutes)	*37.40*	26.99	47.82	20.86	<0.0001
Actigraphy—diary					
TST (minutes)	−*44.90*	−51.71	−38.09	13.62	<0.0001
SE (%)	−*8.41*	−9.89	−6.94	2.95	<0.0001
SOL (minutes)	−**2.95**	−6.06	0.17	6.23	0.063
WASO (minutes)	**14.70**	5.26	24.15	18.62	0.002

Bold = No clinically meaningful differences between instruments. Italics = Clinically meaningful differences between instruments.

ICCs between scEEG, actigraphy, and sleep diary for the averaged sleep parameters are shown in Table 4. Relative to other parameters, TST measurements showed the greatest agreement between instruments for all six nights, with moderate agreement between scEEG vs. actigraphy (ICC = 0.694), poor agreement between scEEG vs. diary (ICC = 0.472), and moderate agreement between actigraphy vs. diary (ICC = 0.584). However, for SE, SOL, and WASO, the instruments consistently showed weak agreement (ICCs < 0.5). All ICCs were significantly different from zero (*p* < 0.05).

**Table 4.** Intraclass correlation coefficients based on average of all available nights from all participants

Comparison	ICC (95% CI)	*F*(df)	*P*
Total sleep time			
scEEG vs. actigraphy	0.694 (0.595–0.766)	*F*(292,292) 3.47	<0.0001
scEEG vs. diary	0.472 (−0.057–0.707)	*F*(292,292) 2.74	<0.0001
Actigraphy vs. diary	0.584 (0.177–0.761)	*F*(292,292) 3.21	<0.0001
Sleep efficiency			
scEEG vs. actigraphy	0.456 (0.316–0.567)	*F*(292,292) 1.87	<0.0001
scEEG vs. diary	0.194 (−0.046–0.378)	*F*(292,292) 1.40	0.002
Actigraphy vs. diary	0.137 (−0.052–0.297)	*F*(292,292) 1.23	0.040
Sleep onset latency			
scEEG vs. actigraphy	0.428 (0.282–0.545)	*F*(292,292) 1.78	<0.0001
scEEG vs. diary	0.479 (0.322–0.598)	*F*(288,288) 2.04	<0.0001
Actigraphy vs. diary	0.333 (0.161–0.470)	*F*(288,288) 1.50	0.0003
Wake after sleep onset			
scEEG vs. actigraphy	0.276 (0.074–0.433)	*F*(292,292) 1.48	0.0005
scEEG vs. diary	0.159 (−0.036–0.320)	*F*(288,288) 1.22	0.045
Actigraphy vs. diary	0.181 (−0.026–0.347)	*F*(288,288) 1.23	0.041

*p < 0.05; **p < 0.01;***p < 0.0001.

**Figure 1.**
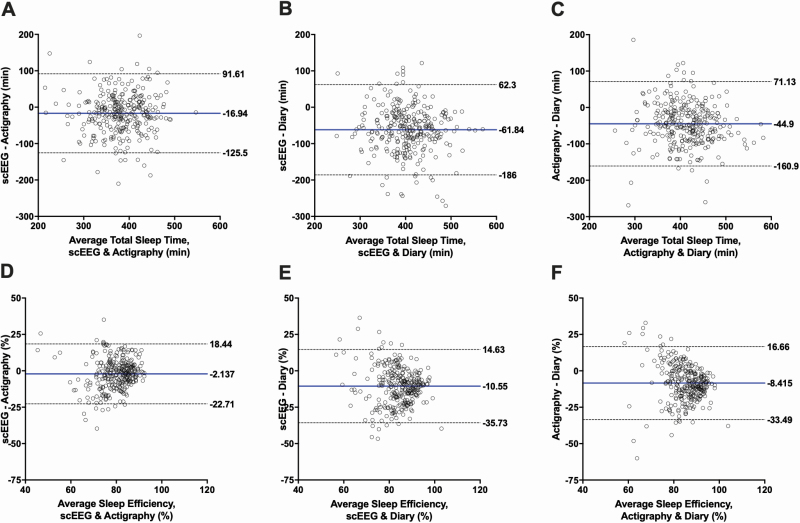
Bland-Altman plots. Each graph shows the comparison between the average total sleep time (A–C) and sleep efficiency (D–F) (x-axis) and the difference in total sleep time and sleep efficiency (y-axis) measured by two instruments. The blue line represents the mean bias between the instruments. The dotted lines denote the 95% limits of agreement. Each row represents a different sleep parameter in comparing single-channel EEG (scEEG) and actigraphy, scEEG and diary, and actigraphy and diary. Axes are standardized across rows. A–C: Total sleep time (TST). D–F: Sleep efficiency (SE).

**Figure 2.**
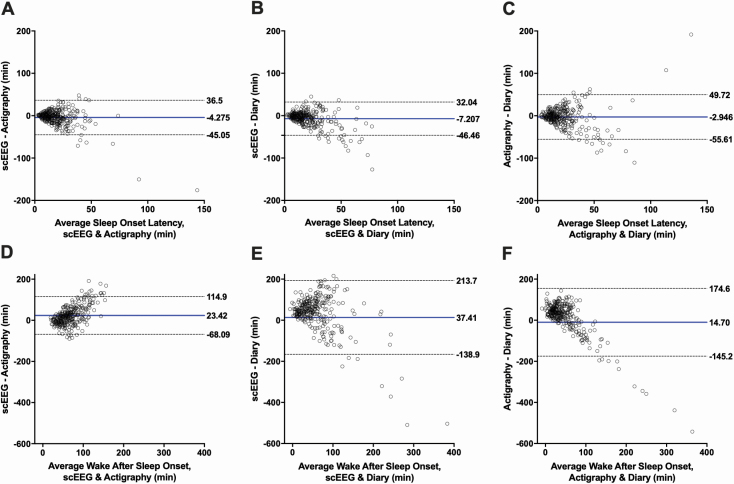
Bland-Altman plots. Each graph shows the comparison between the average sleep onset latency (A–C) and wake after sleep onset (D–F) (x-axis) and the difference in sleep onset latency and wake after sleep onset (y-axis) measured by two instruments. The blue line represents the mean bias between the instruments. The dotted lines denote the 95% limits of agreement. Each row represents a different sleep parameter in comparing single-channel EEG (scEEG) and actigraphy, scEEG and diary, and actigraphy and diary. Axes are standardized across rows. A–C: Sleep Onset Latency (SOL). D–F: Wake After Sleep Onset (WASO).

We further assessed inter-instrument agreement for each sleep parameter using Bland-Altman plots, with the mean difference denoted with a solid blue line and the 95% limits of agreement marked by dashed lines (Figures 1 and 2). Limits of agreement appeared to be wide across all plots. For each sleep parameter, 95% limits of agreement were similarly wide between instrument comparisons, except for WASO, which demonstrated much wider 95% limits for diary comparisons. The most striking observation from these plots is that the bias between instruments increased with indicators of worsening sleep: the data distribution widened with worsening SE <85%, SOL > 20, WASO > 60 minutes for all instrument comparisons. This pattern of increasing bias with worsening sleep was not apparent for TST.

### Effect of cognitive status on agreement between instruments

We compared instrument agreement between CDR and MMSE groups. CDR is the gold standard for defining cognitive status in AD, but MMSE is frequently used in previous studies comparing different instruments that measure sleep. When assessed by *t*-test, the majority of instrument comparisons for TST, SE, SOL, and WASO were statistically different, especially in the CDR 0 and MMSE ≥27 groups (Table 5). The mean differences for all four parameters in CDR 0 and MMSE ≥27 participants had sufficiently narrow 95% CI that scEEG and actigraphy had no clinically significant differences (Table 5). However, for CDR 0.5 and MMSE <27 individuals, the 95% CI widened for all parameters such that there were clinically significant differences in TST, SE, and WASO for scEEG vs. actigraphy. For the diary comparisons, 2 out of 4 parameters in CDR 0 and MMSE ≥27 groups had clinically significant differences, whereas 3 out of 4 parameters in CDR 0.5 and MMSE <27 groups had clinically significant differences. The mean differences were overall greater in magnitude in the CDR 0.5 and MMSE <27 groups. Of the instrument comparisons deemed to have no clinically significant differences, only six mean differences were not statistically significant: SOL (scEEG vs. actigraphy in the CDR 0 and MMSE ≥27 groups; scEEG vs. diary in CDR 0.5 and MMSE <27 groups; actigraphy vs. diary in CDR 0.5 group) and WASO (actigraphy vs. diary in CDR 0.5 group).

**Table 5.** Mean differences and paired *t*-tests for cognitive and AD biomarker groups

		95% CI of mean difference					95% CI of mean difference			
	Mean difference	Lower	Upper	95% CI range	*P*	Mean difference	Lower	Upper	95% CI range	*P*
	*CDR 0*					*CDR 0.5*				
scEEG—actigraphy										
TST (minutes)	−19.30	−23.75	−12.83	**10.92**	<0.0001	−11.64	−34.37	11.09	*45.46*	0.308
SE (%)	−2.83	−4.11	−1.54	**2.57**	<0.0001	−1.42	−5.63	2.80	*8.43*	0.501
SOL (minutes)	−2.11	−4.82	0.60	**5.42**	0.127	−9.86	−16.54	−3.18	**13.36**	0.005
WASO (minutes)	22.39	16.51	28.27	**11.76**	<0.0001	18.03	1.48	34.58	*33.10*	0.033
scEEG—diary										
TST (minutes)	*−55.76*	−63.69	−47.84	15.85	<0.0001	*−84.09*	−103.38	−64.80	38.58	<0.0001
SE (%)	*−10.27*	−12.00	−8.53	3.47	<0.0001	*−13.76*	−18.12	−9.41	8.71	<0.0001
SOL (minutes)	**−7.65**	−10.25	−5.04	5.21	<0.0001	**−2.66**	−6.78	1.46	8.24	0.199
WASO (minutes)	*34.71*	22.34	47.08	24.74	<0.0001	*41.65*	11.36	71.95	60.59	0.008
Actigraphy—diary										
TST (minutes)	*−37.51*	−45.22	−29.80	15.42	<0.0001	*−70.51*	−87.27	−53.75	33.52	<0.0001
SE (%)	*−7.57*	−9.28	−5.86	3.42	<0.0001	*−12.08*	−15.90	−8.27	7.63	<0.0001
SOL (minutes)	**−5.64**	−9.47	−1.80	7.67	0.004	**7.16**	−0.24	14.56	14.8	0.058
WASO (minutes)	**13.25**	2.07	24.44	22.37	0.020	*25.13*	−1.74	52.00	53.74	0.066
	*MMSE ≥27*					*MMSE <27*				
scEEG—actigraphy										
TST (minutes)	−21.54	−28.18	−14.90	**13.28**	<0.0001	15.63	−14.36	45.63	*59.99*	0.291
SE (%)	−3.14	−4.44	−1.83	**2.61**	<0.0001	3.39	−2.44	9.23	*11.67*	0.241
SOL (minutes)	−2.39	−5.05	0.26	**5.31**	0.077	−15.93	−24.08	−7.78	**16.30**	0.001
WASO (minutes)	23.96	18.11	29.81	**11.70**	<0.0001	−1.06	−23.73	21.61	*45.34*	0.924
scEEG—diary										
TST (minutes)	*−59.68*	−67.66	−51.70	15.96	<0.0001	*−73.72*	−99.49	−47.94	51.55	<0.0001
SE (%)	*−10.63*	−12.38	−8.88	3.50	<0.0001	*−12.49*	−17.76	−7.21	10.55	<0.0001
SOL (minutes)	**−7.51**	−10.01	−5.00	5.01	<0.0001	**−3.19**	−8.41	2.04	10.45	0.217
WASO (minutes)	*36.21*	23.96	48.46	24.50	<0.0001	*33.11*	4.46	61.75	57.29	0.026
Actigraphy—diary										
TST (minutes)	*−38.14*	−45.34	−30.94	14.40	<0.0001	*−89.35*	−113.85	−64.84	49.01	<0.0001
SE (%)	*−7.50*	−9.11	−5.88	3.23	<0.0001	*−15.88*	−21.17	−10.59	10.58	<0.0001
SOL (minutes)	**−5.04**	−8.67	−1.42	7.25	0.007	**13.47**	3.61	23.33	19.72	0.010
WASO (minutes)	**12.80**	1.87	23.73	21.86	0.022	*42.39*	13.90	70.88	56.98	0.006
	*Low p-tau/Aβ42*					*High p-tau/Aβ42*				
scEEG—actigraphy										
TST (minutes)	−27.15	−37.82	−16.47	**21.35**	<0.0001	−28.03	−45.82	−10.23	**35.59**	0.003
SE (%)	−4.18	−6.35	−2.01	**4.34**	<0.0001	−4.43	−7.57	−1.29	*6.28*	0.007
SOL (minutes)	0.09	−3.10	3.27	**6.37**	0.957	−3.76	−8.84	1.33	**10.17**	0.144
WASO (minutes)	29.31	19.55	39.08	**19.53**	<0.0001	31.82	18.15	45.50	**27.35**	<0.0001
scEEG—diary										
TST (minutes)	*−73.04*	−86.21	−59.86	26.35	<0.0001	*−63.92*	−82.48	−45.37	37.11	<0.0001
SE (%)	*−12.52*	−15.58	−9.46	6.12	<0.0001	*−10.83*	−14.43	−7.23	7.20	<0.0001
SOL (minutes)	**−4.50**	−8.52	−0.48	8.04	0.029	**−5.55**	−10.07	−1.03	9.04	0.017
WASO (minutes)	*44.23*	18.65	69.81	51.16	0.001	*40.75*	15.10	66.40	51.30	0.003
Actigraphy—diary										
TST (minutes)	*−45.49*	−57.01	−33.98	23.03	<0.0001	*−38.71*	−54.26	−23.16	31.10	<0.0001
SE (%)	*−8.23*	−11.12	−5.36	5.76	<0.0001	*−6.97*	−10.14	−3.80	6.34	<0.0001
SOL (minutes)	**−5.12**	−10.29	0.05	10.34	0.052	**−1.71**	−7.93	4.52	12.45	0.583
WASO (minutes)	**16.36**	−7.50	40.21	47.71	0.175	**9.12**	−13.92	32.16	46.08	0.429

*N* = number of participants in selected cognitive group. Bold = No clinically meaningful differences between instruments. Italics = Clinically significant differences between instruments.

ICCs were generally greater for TST, SE, SOL, and WASO in the CDR 0 and the MMSE ≥27 groups compared to the CDR 0.5 and MMSE <27 groups (Table 6). However, the 95% CIs overlapped between CDR and MMSE groups. For TST, all ICC comparisons between groups and instruments were significantly different from zero. Also, ICCs for scEEG vs. actigraphy exceeded 0.7 in cognitively normal participants for TST. For SE, in the CDR 0.5 and MMSE <27 groups, ICCs were not significantly different from zero for all instrument comparisons. SOL and WASO were found to have variable results depending on the instrument comparisons. For instance, the ICCs for scEEG vs. actigraphy measurements of SOL were significantly different from zero in the CDR 0 and CDR 0.5 groups but not the MMSE <27 group (Table 6). In contrast, SOL comparisons between scEEG vs. diary were greater in the CDR 0.5 and MMSE <27 groups. For WASO, only the ICCs for scEEG vs. actigraphy comparison in the CDR 0 and MMSE groups were significantly different from zero.

**Table 6.** Intraclass correlation coefficients based on average of all available nights by group

Group	CDR 0	CDR 0.5	MMSE ≥ 27	MMSE < 27	Low p-tau/Aβ42	High p-tau/Aβ42
	ICC (95% CI)	ICC (95% CI)	ICC (95% CI)	ICC (95% CI)	ICC (95% CI)	ICC (95% CI)
	*F*(df), *P*	*F*(df), *P*	*F*(df), *P*	*F*(df), *P*	*F*(df), *P*	*F*(df), *P*
Total sleep time						
scEEG vs. actigraphy	0.780 (0.660–0.851)	0.488 (0.061–0.721)	0.731 (0.584–0.817)	0.705 (0.316–0.874)	0.760 (0.456–0.878)	0.564 (0.210–0.760)
	*F*(191,191) 5.14,	*F*(42,42) 1.96,	*F*(211,211) 4.22,	*F*(22,22) 3.41,	*F*(63,63) 5.40,	*F*(44,44) 2.53,
	<0.0001	0.016	<0.0001	0.003	<0.0001	0.001
scEEG vs. diary	0.520 (−0.048–0.749)	0.472 (−0.221–0.771)	0.479 (−0.080–0.720)	0.605 (−0.212–0.862)	0.427 (−0.211–0.729)	0.521 (−0.144–0.784)
	*F*(191,191) 3.12,	*F*(42,42) 3.48,	*F*(211,211) 2.86,	*F*(22,22) 4.81,	*F*(63,63) 3.11,	*F*(44,44) 3.31,
	<0.0001	<0.0001	<0.0001	0.0003	<0.0001	<0.0001
Actigraphy vs. diary	0.628 (0.277–0.784)	0.489 (−0.215–0.780)	0.632 (0.256–0.792)	0.414 (−0.233–0.764)	0.599 (−0.043–0.818)	0.716 (0.265–0.870)
	*F*(191,191) 3.47,	*F*(42,42) 3.54,	*F*(211,211) 3.60,	*F*(22,22) 3.43,	*F*(63,63) 3.93,	*F*(44,44) 4.87,
	<0.0001	<0.0001	<0.0001	0.003	<0.0001)	<0.0001
Sleep efficiency						
scEEG vs. actigraphy	0.550 (0.395–0.664)	0.236 (−0.418–0.587)	0.500 (0.334–0.623)	0.423 (−0.325–0.752)	0.527 (0.207–0.717)	0.398 (−0.041–0.660)
	*F*(191,191) 2.32,	*F*(42,42) 1.31,	*F*(211,211) 2.10,	*F*(22,22) 1.75,	*F*(63,63) 2.36,	*F*(44,44) 1.76,
	<0.0001	0.195	<0.0001	0.099	0.0004	0.033
scEEG vs. diary	0.202 (−0.061–0.403)	0.116 (−0.224–0.416)	0.183 (−0.063–0.375)	0.109 (−0.313–0.493)	0.187 (−0.154–0.456)	0.161 (−0.204–0.460)
	*F*(191,191) 1.42,	*F*(42,42) 1.25,	*F*(211,211) 1.37,	*F*(22,22) 1.24,	*F*(63,63) 1.46,	*F*(44,44) 1.36,
	0.008	0.234	0.011	0.306	0.069	0.156
Actigraphy vs. diary	0.179 (−0.054–0.365)	0.256 (−0.179–0.560)	0.232 (−0.008–0.416)	0.048 (−0.243–0.386)	0.138 (−0.214–0.417)	0.383 (−0.066–0.652)
	*F*(191,191) 1.30,	*F*(42,42) 1.66,	*F*(211,211),	*F*(22,22) 1.13,	*F*(63,63) 1.24,	*F*(44,44) 1.88,
	0.035	0.052	0.006	0.387	0.198	0.020
Sleep onset latency						
scEEG vs. actigraphy	0.482 (0.313–0.609)	0.374 (−0.087–0.649)	0.493 (0.336–0.612)	0.194 (−0.340–0.584)	0.610 (0.356–0.764)	0.531 (0.158–0.740)
	*F*(191,191) 1.94,	*F*(42,42) 1.69,	*F*(211,211) 1.98,	*F*(22,22) 1.40,	*F*(63,63) 2.54,	*F*(44,44) 2.16,
	<0.0001	0.046	<0.0001	0.217	0.0002	0.006
scEEG vs. diary	0.508 (0.306–0.647)	0.764 (0.561–0.873)	0.542 (0.362–0.667)	0.623 (0.080–0.849)	0.667 (0.451–0.799)	0.552 (0.198–0.752)
	*F*(189,189) 2.22,	*F*(40,40) 4.29,	*F*(210,210) 2.38,	*F*(19,19) 2.70,	*F*(62,62) 3.15,	*F*(43,43) 2.36,
	<0.0001	<0.0001	<0.0001	0.018	<0.0001	0.003
Actigraphy vs. diary	0.219 (−0.029–0.409)	0.409 (−0.068–0.679)	0.269 (0.048–0.440)	−0.044 (−0.871–0.507)	0.313 (−0.110–0.579)	0.520 (0.115–0.739)
	*F*(189,189) 1.29,	*F*(40,40) 1.74,	*F*(210,210) 1.38,	*F*(19,19) 0.942,	*F*(62,62) 1.48,	*F*(43,43) 2.06,
	0.040	0.042	0.010	0.551	0.063	0.010
Wake after sleep onset						
scEEG vs. actigraphy	0.386 (0.140–0.556)	0.058 (−0.621–0.0469)	0.351 (0.105–0.525)	0.195 (−0.997–0.665)	0.469 (0.022–0.702)	0.146 (−0.290–0.471)
	*F*(191,191) 1.80,	*F*(42,42) 1.07,	*F*(211,211) 1.71,	*F*(22,22) 1.23,	*F*(63,63) 2.37,	*F*(44,44) 1.25,
	<0.0001	0.417	<0.0001	0.315	0.0004	0.235
scEEG vs. diary	0.158 (−0.086–0.351)	−0.031 (−0.718–0.411)	0.122 (−0.114–0.312)	0.038 (−0.881–0.569)	0.194 (−0.237–0.489)	0.050 (−0.527–0.438)
	*F*(189,189) 1.22,	*F*(40,40) 0.965,	*F*(210,210) 1.16,	*F*(19,19) 1.05,	*F*(62,62) 1.28,	*F*(43,43) 1.06,
	0.09	0.544	0.141	0.459	0.165	0.421
Actigraphy vs. diary	0.186 (−0.077–0.385)	0.118 (−0.578–0.517)	0.188 (−0.059–0.378)	−0.004 (−0.733–0.513)	0.224 (−0.271–0.528)	0.028 (−0.793–0.472)
	*F*(189,189) 1.23,	*F*(40,40) 1.14,	*F*(210,210) 1.24,	*F*(19,19) 0.995,	*F*(62,62) 1.29,	*F*(43,43) 1.03,
	0.075	0.339	0.063	0.505	0.157	0.463

Finally, we tested if there were group differences within instruments for TST, SE, SOL, and WASO. For scEEG, there were no statistically significant group differences between CDR or MMSE groups (Table 7). For actigraphy, SE, SOL, and WASO were found to have significant mean differences between CDR and MMSE groups. In contrast, for the sleep diary, only TST was significantly different between CDR and MMSE groups.

**Table 7.** Group differences in sleep measures within devices

Group	CDR 0 vs. CDR 0.5		MMSE ≥27 vs. MMSE <27		Low vs. High p-tau/Aβ42	
	*F*(df)	*P*	*F*(df)	*P*	*F*(df)	*P*
scEEG						
TST	*F*(1,233) 0.042	0.837	*F*(1,233) 3.413	0.066	*F*(1,107) 0.716	0.399
SE	*F*(1,233) 1.152	0.284	*F*(1,233) 0.115	0.735	*F*(1,107) 0.237	0.627
SOL	*F*(1,233) 0.421	0.517	*F*(1,233) 0.064	0.801	*F*(1,107) 0.246	0.621
WASO	*F*(1,233) 0.476	0.491	*F*(1,233) 0.394	0.531	*F*(1,107) 0.097	0.756
Actigraphy						
TST	*F*(1,233) 0.115	0.735	*F*(1,233) 1.106	0.294	*F*(1,107) 0.866	0.354
SE	*F*(1,233) 3.873	0.050	*F*(1,233) 10.862	0.001	*F*(1,107) 0.440	0.508
SOL	*F*(1,233) 5.78	0.017	*F*(1,233) 8.357	0.004	*F*(1,107) 0.839	0.362
WASO	*F*(1,233) 3.987	0.047	*F*(1,233) 13.916	0.00024	*F*(1,107) 1.354	0.247
Diary						
TST	*F*(1,233) 9.36	0.002	*F*(1,233) 8.84	0.003	*F*(1,107) 0.058	0.810
SE	*F*(1,233) 1.187	0.277	*F*(1,233) 1.276	0.260	*F*(1,107) 0.024	0.878
SOL	*F*(1,229) 1.997	0.159	*F*(1,229) 1.589	0.209	*F*(1,105) 0.018	0.892
WASO	*F*(1,229) 0.105	0.747	*F*(1,229) 0.428	0.514	*F*(1,105) 0.015	0.904

Bland-Altman plots for CDR and MMSE groups demonstrated wider 95% limits of agreement for scEEG vs. actigraphy in CDR 0.5 and MMSE <27 groups, most noticeably for TST and SE (Supplementary Figures 7–14). CDR and MMSE status did not show a consistent pattern of change for the 95% limits for diary comparisons. These results should be interpreted with caution given the large discrepancy in group sizes.

### Effect of AD pathology on agreement between instruments

Statistically significant differences by *t*-test were found between most instrument comparisons in both p-tau/Aβ42 groups (Table 5). No clinically significant differences were found for scEEG vs. actigraphy in the low and high p-tau/Aβ42 groups, except for SE in the high group. However, the 95% CI were wider in the high group than the low group. For the diary comparisons, scEEG vs. diary TST, SE, and WASO, as well as actigraphy vs. diary TST and SE were found to have clinically significant differences in both low and high p-tau/Aβ42 groups. The mean differences between low and high groups were similar.

Unlike the CDR and MMSE groups, ICCs were not generally greater for TST, SE, SOL, and WASO in the low p-tau/Aβ42 group (Table 6). For TST, all ICCs for the three instrument comparisons were significantly different from zero but the 95% CIs overlapped. For SE, scEEG vs. diary comparisons in both low and high p-tau/Aβ42 groups were not significantly different from zero as was the actigraphy vs. diary comparison in the low p-tau/Aβ42 group. For SOL, interestingly, the actigraphy vs. diary comparison in the low p-tau/Aβ42 group was not significantly different from zero in contrast to the high p-tau/Aβ42 group. For WASO, only the ICC for the scEEG vs. actigraphy comparison in the low p-tau/Aβ42 group was significantly different from zero. Finally, testing for group differences within instruments, there were no significant differences for TST, SE, SOL, or WASO between the low and high p-tau/Aβ42 groups (Table 7).

Similar to CDR and MMSE, Bland-Altman plots showed wider 95% limits of agreement for scEEG vs. actigraphy in the high p-tau/Aβ42 group for TST, SE, SOL, and WASO (Supplementary Figures 15–18). For scEEG vs. diary or actigraphy vs. diary, the high p-tau/Aβ42 group showed wider limits for TST, similar limits to the low group for SE and SOL, and narrower 95% limits for WASO.

## Discussion

In this study, we compared sleep parameters obtained via scEEG, actigraphy, and sleep diary in a cognitively normal and mildly symptomatic older adult population. We took a multifaceted approach to assessing instrument agreement. First, we used paired t-tests to assess differences between instruments and found that most differences were statistically significant. However, using a published criteria defining clinically significant differences when comparing sleep parameters between different instruments via paired *t*-tests [47], we found that differences between scEEG and actigraphy were not clinically significant. This suggests that the apparent differences between scEEG and actigraphy are consistent enough that these two instruments can potentially be used to measure TST, SE, SOL, and WASO after accounting for their biases in a clinical setting for diagnostic purposes. If greater precision is necessary in a research setting, these differences may not be permissible. We found that sleep diaries may provide similar measurements to either scEEG or actigraphy for SOL and WASO but not for TST and SE, suggesting that sleep diaries capture different aspects of these sleep parameters. Perceived sleep quality, though not formally assessed in our participants, certainly influences sleep diary entries and is an important facet of a comprehensive clinical sleep evaluation. The discrepancy between objective and subjective methods is consistent with patterns in previous studies and may reflect the observation that objective sleep assessment poorly predicts subjective sleep quality [27–34]. This discrepancy between objective sleep measures, such as polysomnography, and subjective sleep quality has led to the suggestion that polysomnography should not be considered the gold standard for sleep measurement [35]. Different sleep measurement methods may capture different aspects of sleep quality and argue for using a multi-modal approach for assessing sleep-wake activity.

We also found that TST overall had the greatest agreement between instruments, although the ICCs ranged from 0.694 for scEEG vs. actigraphy comparisons (moderate agreement) to 0.472 for scEEG vs. diary comparisons (poor agreement). SE, SOL, and WASO showed poor agreement as measured by ICC. Previous work in young to middle-aged adults comparing actigraphy and sleep diaries has also found TST to be more strongly correlated between instruments than parameters such as SE, SOL, and WASO [28, 36]. In a previous study, we compared the scEEG to PSG and found excellent reliability between the measures for TST (ICC = 0.96), good reliability for SE (ICC = 0.86) and WASO (ICC = 0.79), and moderate reliability for SOL (ICC = 0.67) [20]. Although we did not compare actigraphy and sleep diaries to PSG in this study, our previous study suggests that scEEG is an excellent approximation of TST, SE, and WASO as measured by PSG.

Consistent with studies in other populations [20, 32, 34, 55, 56], Bland-Altman plots identified that quantitative markers of worsened sleep, such as lower SE, higher SOL, and higher WASO, resulted in increased disagreement between instruments. Additionally, we observed wide 95% limits of agreement for all sleep measures, which may not be acceptable for research and/or clinical use. Other studies comparing objective and subjective instruments via this method of analysis have generally deemed that agreement is poor, but interpretation of results has been variable despite similar limits of agreement compared to our findings [27, 34, 36, 37].

Our study also suggests that cognitive impairment as assessed by CDR or MMSE and the presence of AD pathology measured by the CSF p-tau/Aβ42 ratio worsens the agreement between scEEG and actigraphy. Separating our participants by CDR or MMSE status demonstrated a trend of worsening statistical and clinical agreement in mildly symptomatic individuals, but this was not consistent across all sleep parameters and instrument comparisons. This pattern was most prominently seen for comparisons of scEEG vs. actigraphy and for measurements of TST. Most ICCs were greater in CDR 0 and MMSE ≥27 groups, but the overlapping CIs temper this observation. 95% limits of agreement from Bland-Altman plots were wider for TST and SE in scEEG vs. actigraphy for CDR 0.5 and MMSE <27 subgroups, but the small size of these subgroups may limit this interpretation. The groups with and without biomarker evidence of AD pathology (i.e. low and high p-tau/Aβ42 ratio) did not show a clear trend in clinical agreement or ICCs, but the high p-tau/Aβ42 group did have wider 95% limits of agreement from Bland-Altman plots for all four sleep parameters in comparing scEEG vs. actigraphy. It is important to note the greater sample size imbalance between CDR and MMSE groups compared to the p-tau/Aβ42 groups. Thus, it may be necessary to exercise caution in comparing scEEG to actigraphy in older adults with cognitive impairment and/or evidence of AD pathology. It may be that scEEG and actigraphy provide different information about sleep-wake activity in mildly impaired adults. Some speculation as to the etiology of this phenomenon include changes on sleep EEG architecture that are linked to the presence of AD pathology [44] that would not be captured on actigraphy, as well as the finding that actigraphy may be less accurate in older adults with sleep disturbances due to motionless wake being scored incorrectly as sleep [57].

For the sleep diary, clinical agreement for the various sleep parameters was already limited for CDR 0, MMSE ≥27, and low p-tau/Aβ42, and differences between instruments appeared to worsen slightly for CDR 0.5 and MMSE <27 groups, but not for the high p-tau/Aβ42 group. Cognitively impaired groups or the high p-tau/Aβ42 group was associated with lower ICCs and/or ICCs not significantly different from zero. The 95% limits of agreement via Bland-Altman plots did not show a consistent effect of CDR, MMSE, or p-tau/Aβ42 across different sleep parameters in diary comparisons. These results hint that cognitive status or biomarker evidence of AD pathology may worsen the agreement of the sleep diary with scEEG or actigraphy, but lack of consistently worsened agreement across multiple analyses and multiple parameters makes this less compelling. This may reflect the already inherent limitations of sleep diaries in providing accurate numeric measurements of sleep, independent of cognitive status or presence of AD pathology. Sleep diaries are prone to recall bias, may be filled out by persons other than the participant, and require multiple days of sustained adherence.

In general, prior work on the effect of cognition on sleep instrument agreement found that impaired cognition decreases agreement between subjective and objective instruments. A study of over 900 older adults found that cognitive function assessed by the MMSE was associated with decreased agreement between actigraphy and diary-measured TST [58]. Other studies have been limited by small sample sizes, such as one study comparing PSG and sleep diaries in 25 cognitively normal adults and 25 mildly impaired adults and found that only cognitively normal adults had significant relationships between slow-wave sleep arousals on PSG and self-reported sleep disturbances [38]. Further, it was found that mildly impaired patients significantly misperceived SOL in comparing self-report to PSG whereas cognitively normal adults did not. In another study, 55 people with early to moderate-stage AD were compared to 26 cognitively normal older adults and found that sleep questionnaires poorly predicted actigraphic results in both groups [40]. However, MMSE scores did not correlate with subjective-objective differences suggesting that the discrepancy was not related to cognitive functioning as assessed by the MMSE [40]. Another study in 59 older adults with cognitive impairment and depression used an extensive battery of cognitive tests (including MMSE, CDR, and other dementia-specific tests) and found that delayed memory, but not level of executive functioning, was associated with increased discrepancy between actigraphy and the Pittsburgh Sleep Quality Index [39]. However, there have been several other studies that showed no effect of cognition as measured by MMSE or the Montreal Cognitive Assessment [31, 33].

Several previous studies did not find that MMSE score affected agreement between sleep parameters measured by different instruments [31, 33, 40], possibly due to smaller sample sizes (<100 participants). The mixed results between the MMSE groups in this study and previous studies signify the limited utility of the MMSE as a screening tool compared to a comprehensive cognitive assessment. The CDR collects significantly more information about multiple domains of daily living to assess cognitive function compared to the MMSE, which is a one-time battery of short neuropsychologic tests. Although the MMSE can be sensitive in detecting overt dementia, it does not discriminate as well between CDR 0 and 0.5 [59], has a limited role in identifying mildly impaired individuals if only administered once or in isolation of other tests [60, 61], and can be affected by education and age [51].

Our study demonstrated differences in agreement between scEEG and actigraphy by cognitive status and biomarker evidence of AD pathology. scEEG and actigraphy showed reasonable agreement for TST, SE, SOL, and WASO in cognitively normal older adults, with the best agreement observed for TST. Caution should be exercised when comparing these methods in mildly symptomatic individuals or those with biomarker evidence of AD pathology. Sleep diaries likely capture different aspects of sleep compared to scEEG and actigraphy and are an important component of a thorough sleep evaluation. Future work will need to further investigate the relationship between sleep instruments and cognitive function and AD pathology, especially using robust cognitive assessments (e.g. CDR) and AD biomarkers (e.g. CSF p-tau/Aβ42).

## Limitations

There are several limitations to our study. First, we used published criteria from an AASM-commissioned task force [47] for comparing clinically significant agreement between actigraphy vs. PSG and actigraphy vs. sleep diary, and extrapolated this criteria to the scEEG device. We considered these criteria to be reasonable, but these may not be acceptable if a good or excellent level of agreement is needed to achieve the necessary precision of measurements by different instruments. Second, the majority of our participants were cognitively normal with ~20% showing evidence of symptomatic AD. Third, only a limited number of patients had CSF data available for analysis. Fourth, individuals who participate in longitudinal biomarker studies of AD almost certainly are not representative of the general population.

## Supplementary Material

zpaa006_suppl_Supplementary_Materials
