# Supplementary material for: Comparison of single-channel EEG, actigraphy, and sleep diary in cognitively normal and mildly impaired older adults
Source: Sleep Adv. 2020 Oct 24;1(1):zpaa006. doi: 10.1093/sleepadvances/zpaa006 (PMC7898727; doi:10.1093/sleepadvances/zpaa006)

Title: Comparison of single-channel EEG, actigraphy, and sleep diary in cognitively normal and mildly impaired older adults.

Authors: Chris A. Chou^1^; Cristina D. Toedebusch^1^; Tiara Redrick^1^; David Freund^1^; Jennifer S. McLeland^1^; John C. Morris^1,2,3^; David M. Holtzman^1,2,3^; and Brendan P. Lucey^1,2*^.

^1^Department of Neurology, Washington University School of Medicine, St Louis, MO

^2^Hope Center for Neurological Disorders, Washington University School of Medicine, St Louis, MO

^3^Knight Alzheimer Disease Research Center, Washington University School of Medicine, St Louis, MO

^*^Corresponding author

Address:

Campus Box 8111

660 South Euclid Avenue

St Louis, MO 63110

Phone: 314-747-3805

Email: luceyb@wustl.edu

**Supplementary Table 1.** Average Sleep Parameters for Individual Nights and Average of All Available Nights

|  | **N1** | **N2** | **N3** | **N4** | **N5** | **N6** | **Average** |
| --- | --- | --- | --- | --- | --- | --- | --- |
| **scEEG** (*n*=199-238) |  |  |  |  |  |  |  |
| TST (min) | 381.4 (79.4) | 375.1 (80.2) | 386.5 (78.7) | 366.7 (76.6) | 375.9 (76.1) | 374.5 (69.9) | 373.7 (59.1) |
| SE (%) | 79.1 (11.9) | 79.2 (11.9) | 79.8 (12.0) | 78.7 (11.8) | 79.3 (10.5) | 79.4 (10.9) | 78.7 (9.3) |
| SOL (min) | 18.5 (17.6) | 16.7 (16.8) | 17.3 (18.8) | 16.6 (15.8) | 15.7 (16.0) | 19.0 (19.0) | 17.6 (12.4) |
| WASO (min) | 77.9 (53.8) | 80.9 (57.5) | 76.2 (53.6) | 79.9 (56.1) | 77.0 (48.2) | 74.1 (50.6) | 80.0 (46.5) |
| **Actigraphy** (*n*=265-290) |  |  |  |  |  |  |  |
| TST (min) | 392.7 (76.5) | 392.3 (78.0) | 395.8 (80.1) | 382.3 (79.2) | 393.7 (77.2) | 384.4 (77.3) | 390.6 (58.0) |
| SE (%) | 81.1 (10.8) | 81.5 (10.0) | 80.7 (11.1) | 80.2 (11.0) | 81.1 (9.5) | 80.3 (10.5) | 80.9 (8.4) |
| SOL (min) | 23.4 (33.9) | 18.5 (26.3) | 22.4 (32.1) | 23.1 (32.6) | 21.1 (29.1) | 23.4 (32.6) | 21.9 (21.1) |
| WASO (min) | 56.6 (29.0) | 57.1 (29.9) | 56.5 (29.3) | 54.6 (30.2) | 57.4 (29.4) | 58.2 (31.6) | 56.6 (23.1) |
| **Diary** (*n*=254-289) |  |  |  |  |  |  |  |
| TST (min) | 435.8 (80.9) | 435.1 (79.5) | 438.1 (89.3) | 435.3 (83.2) | 437.0 (81.9) | 431.4 (82.0) | 435 (63.3) |
| SE (%) | 89.6 (14.7) | 89.5 (15.5) | 89.2 (18.5) | 90.0 (15.0) | 91.2 (13.3) | 88.4 (16.6) | 89.3 (10.5) |
| SOL (min) | 25.8 (34.9) | 24.6 (27.9) | 24.1 (28.3) | 23.8 (31.3) | 23.7 (29.9) | 24.3 (27.7) | 24.7 (21.3) |
| WASO (min) | 35.4 (119.7) | 47.9 (156.2) | 48.6 (156.4) | 42.4 (144.2) | 25.5 (88.8) | 52.4 (160.3) | 41.6 (83.0) |

N1, N2, N3… = night # of data collection. *n* = range of number of participants with available data for specified instrument and parameter on any night. TST = total sleep time. SE = sleep efficiency. SOL = sleep onset latency. WASO = wake after sleep onset.

**Supplementary Table 2**. Number of Participant Sleep Monitoring Nights

| **Total Sleep Monitoring Nights** | **Number of Participants** |
| --- | --- |
| 2 | 22 (7.5%) |
| 3 | 46 (15.7%) |
| 4 | 60 (20.5%) |
| 5 | 73 (24.9%) |
| 6 | 92 (31.4%) |

**Supplementary Table 3.** Pearson Correlations (*r*) for Individual Nights and Average of All Available Nights

| **Comparison** | **N1** | | **N2** | | **N3** | | **N4** | | **N5** | | **N6** | | **Average** | |
| --- | --- | --- | --- | --- | --- | --- | --- | --- | --- | --- | --- | --- | --- | --- |
|  | *n* | *r* | *n* | *r* | *n* | *r* | *n* | *r* | *n* | *r* | *n* | *r* | *n* | *r* |
| **Total sleep time** |  |  |  |  |  |  |  |  |  |  |  |  |  |  |
| scEEG vs. actigraphy | 220 | 0.621*** | 237 | 0.639*** | 224 | 0.640*** | 232 | 0.635*** | 215 | 0.695*** | 197 | 0.639*** | 293 | 0.553*** |
| scEEG vs. diary | 228 | 0.554*** | 235 | 0.557*** | 224 | 0.590*** | 230 | 0.550*** | 215 | 0.499*** | 194 | 0.610*** | 293 | 0.466*** |
| Actigraphy vs. diary | 287 | 0.556*** | 283 | 0.577*** | 286 | 0.579*** | 284 | 0.522*** | 270 | 0.558*** | 253 | 0.546*** | 293 | 0.527*** |
| **Sleep efficiency** |  |  |  |  |  |  |  |  |  |  |  |  |  |  |
| scEEG vs. actigraphy | 220 | 0.291*** | 237 | 0.320*** | 224 | 0.237*** | 232 | 0.336*** | 215 | 0.297*** | 197 | 0.358*** | 293 | 0.304*** |
| scEEG vs. diary | 219 | 0.236*** | 235 | 0.189** | 221 | 0.228** | 229 | 0.166* | 215 | 0.212** | 192 | 0.179* | 293 | 0.168** |
| Actigraphy vs. diary | 286 | 0.168** | 283 | 0.136* | 282 | 0.102 | 283 | 0.160** | 270 | 0.128* | 251 | 0.035 | 293 | 0.105 |
| **Sleep onset latency** |  |  |  |  |  |  |  |  |  |  |  |  |  |  |
| scEEG vs. actigraphy | 220 | 0.334*** | 235 | 0.397*** | 223 | 0.241*** | 232 | 0.301*** | 214 | 0.060 | 196 | 0.291*** | 293 | 0.321*** |
| scEEG vs. diary | 212 | 0.358*** | 229 | 0.456*** | 216 | 0.402*** | 227 | 0.274*** | 211 | 0.271*** | 186 | 0.237** | 289 | 0.392*** |
| Actigraphy vs. diary | 277 | 0.215*** | 274 | 0.207** | 274 | 0.150* | 278 | 0.193** | 261 | 0.102 | 243 | 0.088 | 289 | 0.201** |
| **Wake after sleep onset** |  |  |  |  |  |  |  |  |  |  |  |  |  |  |
| scEEG vs. actigraphy | 220 | 0.261*** | 237 | 0.312*** | 224 | 0.237*** | 232 | 0.298*** | 215 | 0.255*** | 197 | 0.334*** | 293 | 0.241*** |
| scEEG vs. diary | 210 | 0.077 | 229 | 0.164* | 215 | 0.180** | 226 | 0.039 | 210 | 0.192** | 185 | 0.038 | 289 | 0.118* |
| Actigraphy vs. diary | 275 | 0.134* | 274 | 0.174** | 269 | 0.121* | 276 | 0.193** | 260 | 0.122* | 241 | 0.087 | 289 | 0.198** |

N1, N2, N3… = night # of data collection. *n* = number of subjects with available data.

**p*<0.05; ***p*<0.01; ****p*<0.0001.

**Supplementary Figures**

**Supplementary Figure 1. Single-channel EEG (scEEG) vs. actigraphy scatterplot.** Pearson correlations (*r*) and significance level are shown for each scatterplot and group. Axes are standardized for the same sleep parameter (each column). A-D: Composite participant data for total sleep time (TST), sleep efficiency (SE), sleep onset latency (SOL), and wake after sleep onset (WASO).


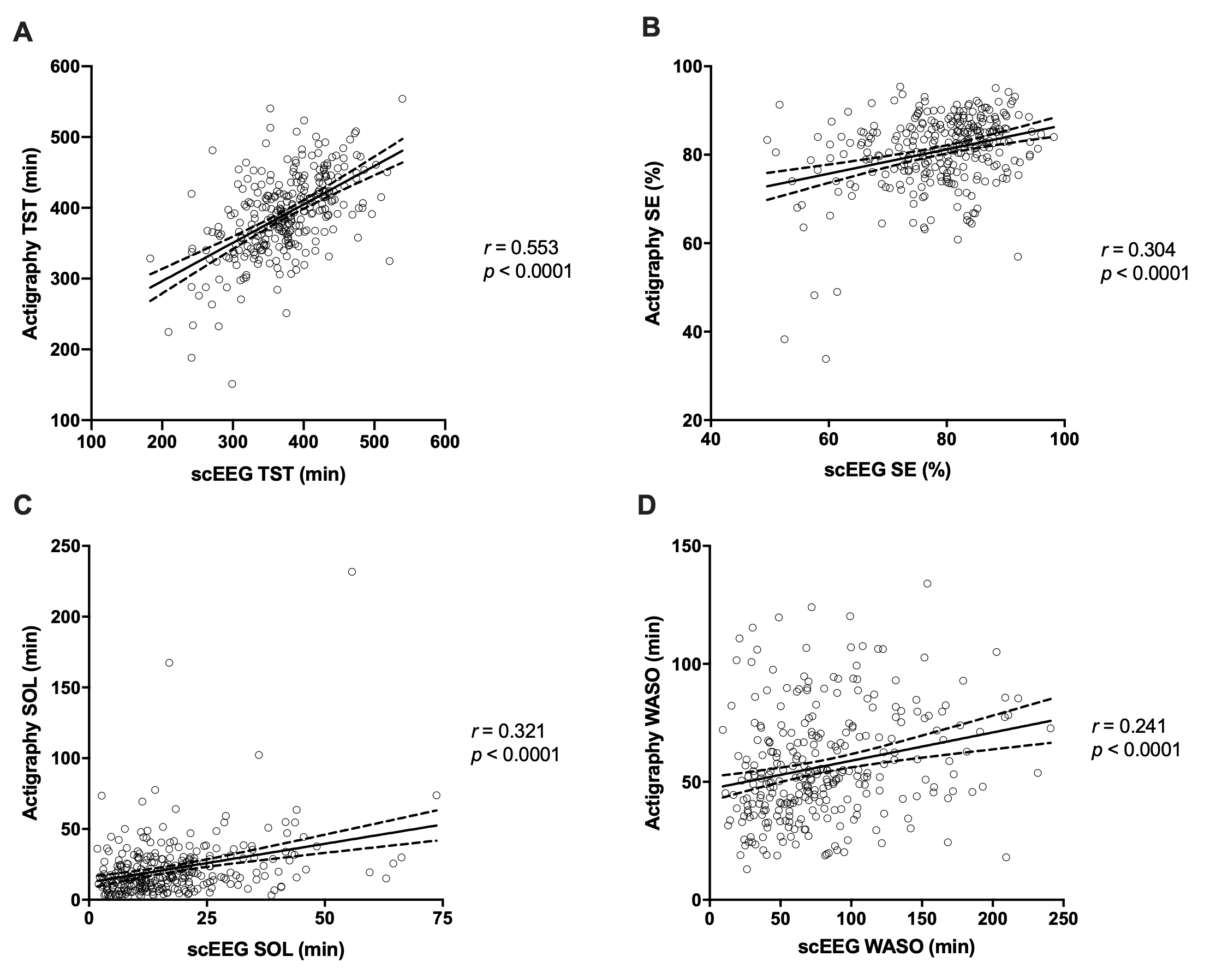


**Supplementary Figure 2. Single-channel EEG (scEEG) vs. sleep diary scatterplot.** Pearson correlations (*r*) and significance level are shown for each scatterplot and group. Axes are standardized for the same sleep parameter (each column). A-D: Composite participant data for total sleep time (TST), sleep efficiency (SE), sleep onset latency (SOL), and wake after sleep onset (WASO).


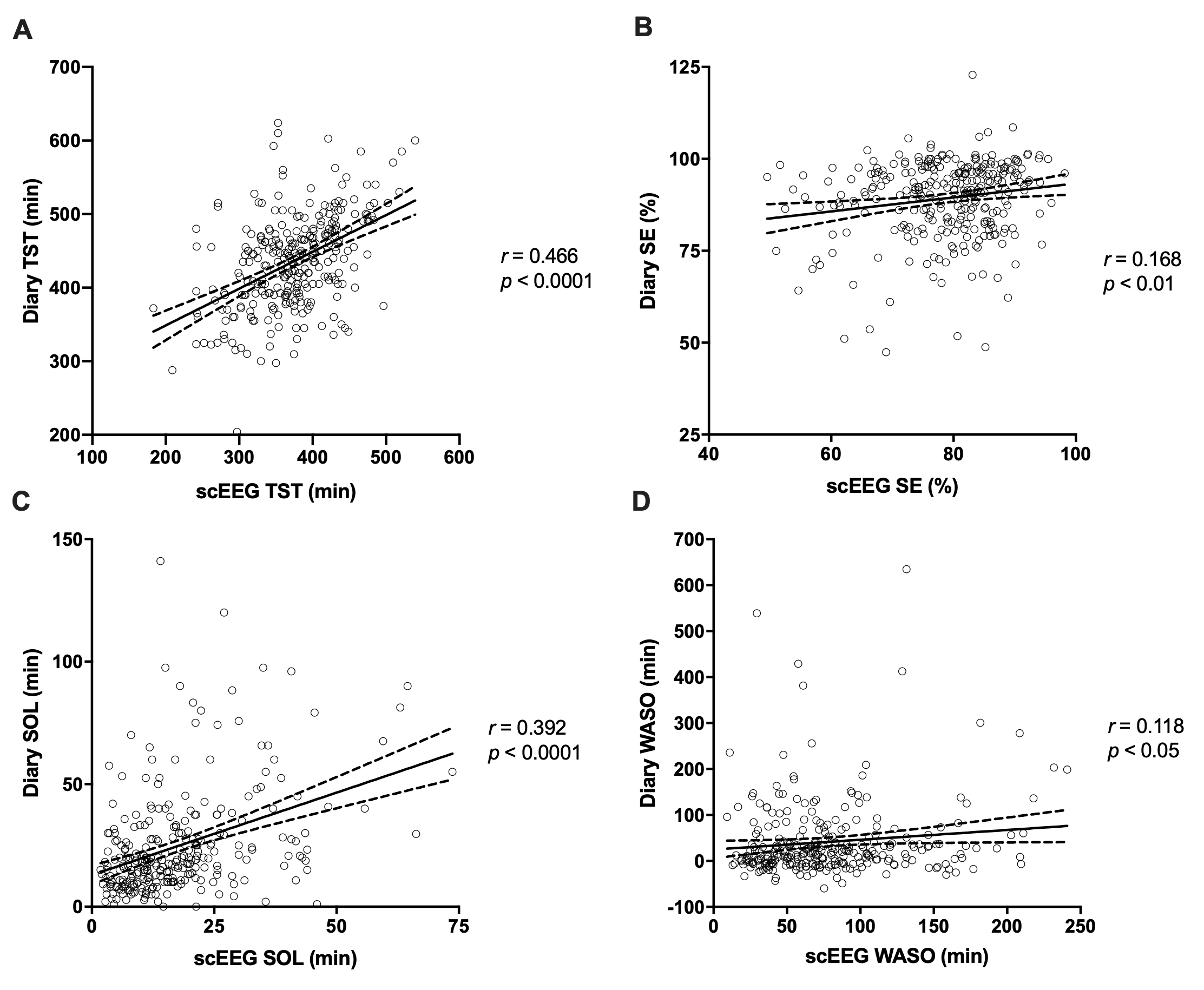


**Supplementary Figure 3. Actigraphy vs. diary scatterplot.** Pearson correlations (*r*) and significance level are shown for each scatterplot and group. Axes are standardized for the same sleep parameter (each column). A-D: Composite participant data for total sleep time (TST), sleep efficiency (SE), sleep onset latency (SOL), and wake after sleep onset (WASO).


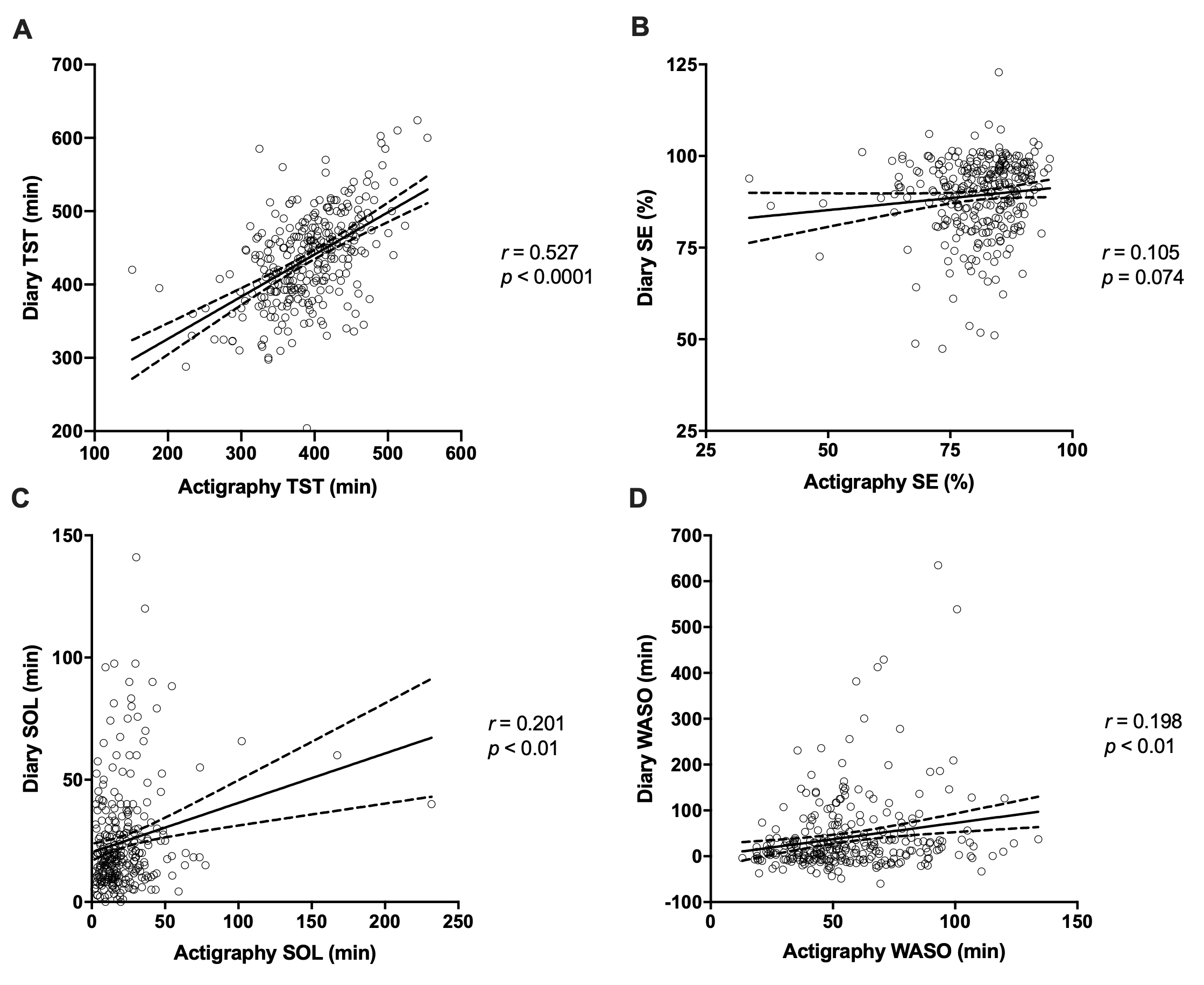


**Supplementary Figure 4. Scatterplots of Clinical Dementia Rating (CDR) groups**. Pearson correlations (*r*) and significance level are shown for each scatterplot and CDR group. Axes are standardized for the same sleep parameter (each column). Participant data separated by CDR 0 (blue) and CDR 0.5 (red) for each sleep parameter. scEEG vs. actigraphy comparisons are shown for TST, SE, SOL, and WASO (A-D), scEEG vs. diary comparisons are shown for TST, SE, SOL, and WASO (E-H), and actigraphy vs. diary comparisons are shown for TST, SE, SOL, and WASO (I-L).


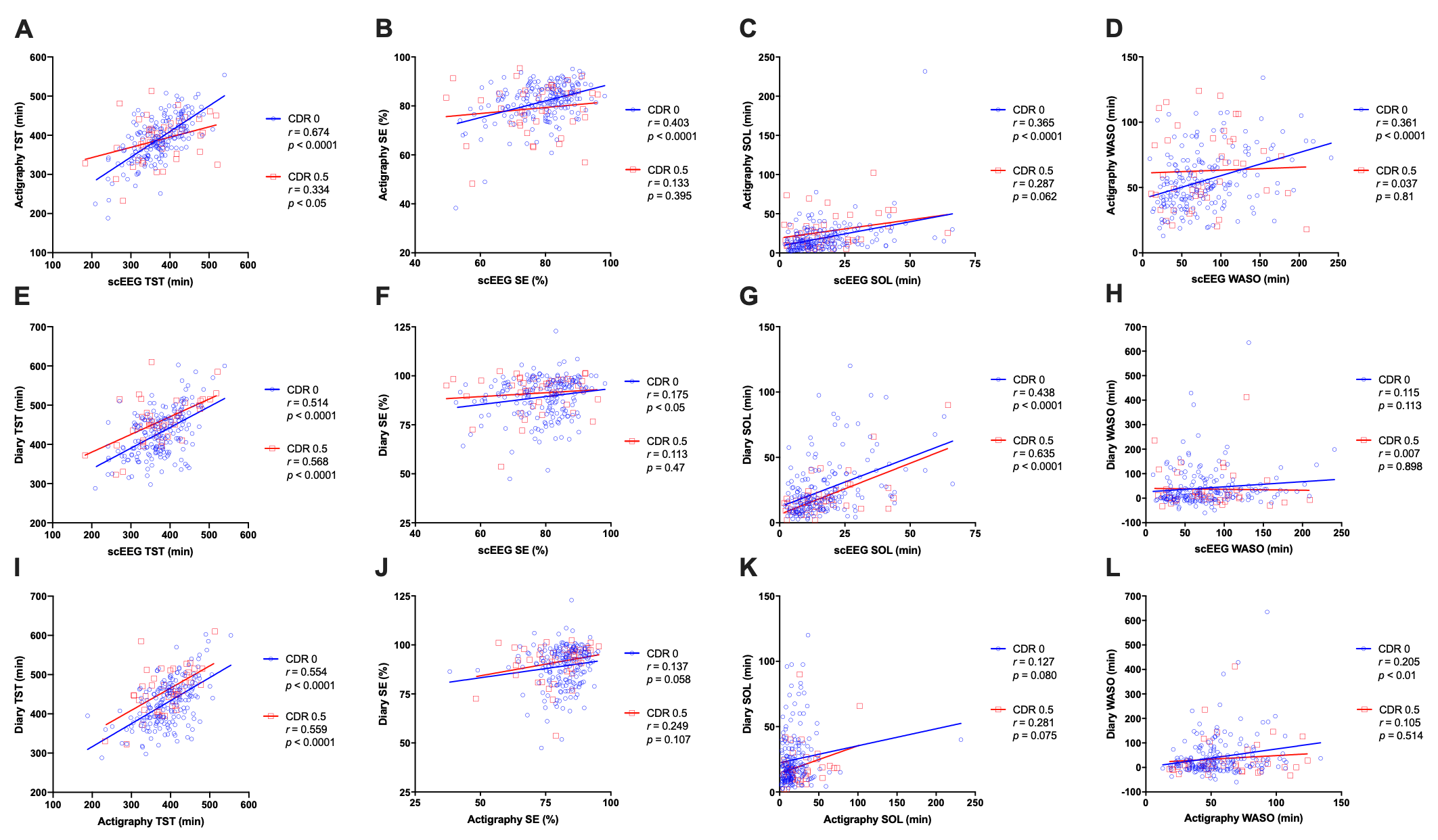


**Supplementary Figure 5. Scatterplots of Mini Mental State Examination (MMSE) groups**. Pearson correlations (*r*) and significance level are shown for each scatterplot and MMSE group. Axes are standardized for the same sleep parameter (each column). Participant data MMSE ≥27 (blue) and MMSE <27 (red) for each sleep parameter. scEEG vs. actigraphy comparisons are shown for TST, SE, SOL, and WASO (A-D), scEEG vs. diary comparisons are shown for TST, SE, SOL, and WASO (E-H), and actigraphy vs. diary comparisons are shown for TST, SE, SOL, and WASO (I-L).


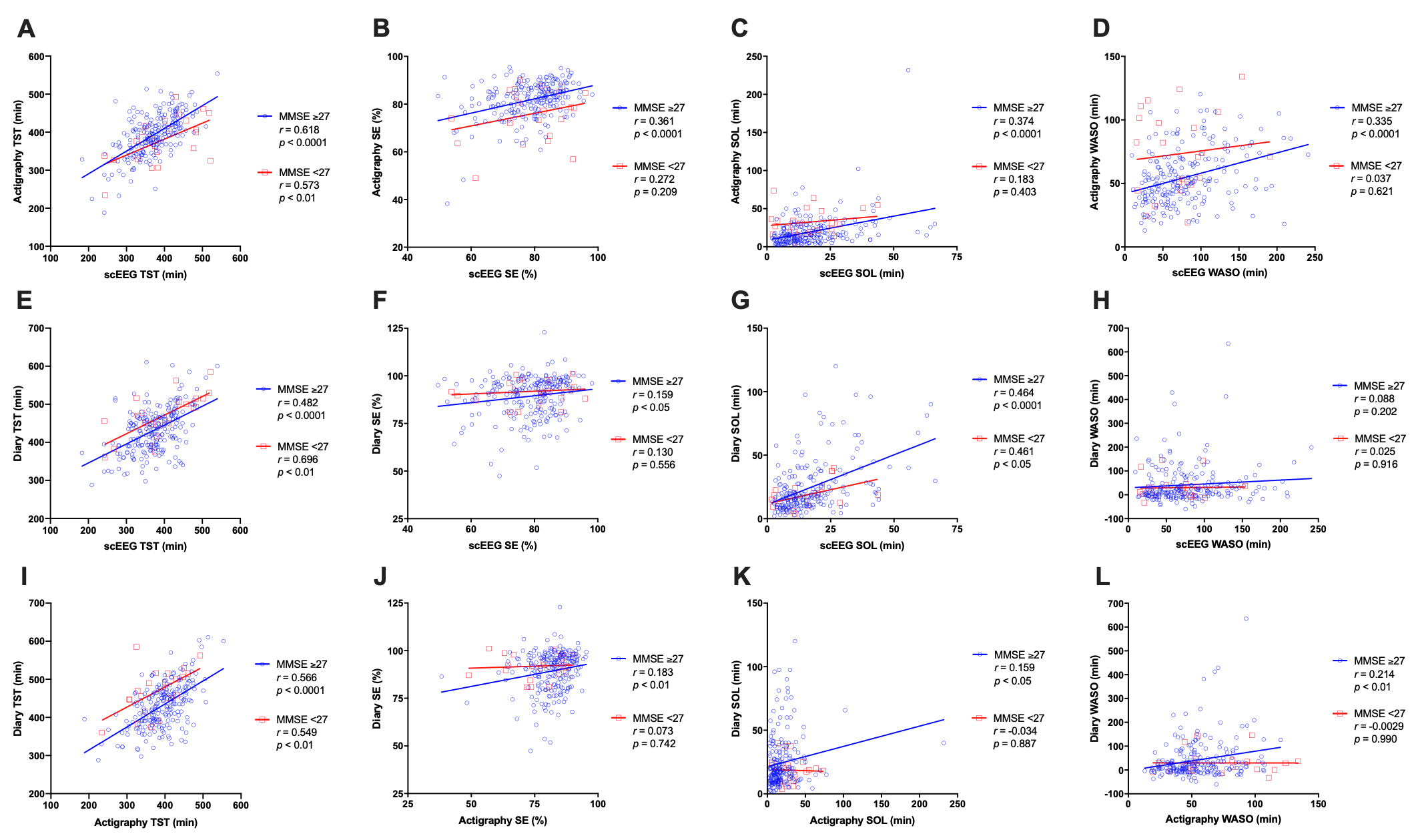


**Supplementary Figure 6. Scatterplots of phosphorylated tau (p-tau)/amyloid-β-42 peptide (Aβ42) groups**. Pearson correlations (*r*) and significance level are shown for each scatterplot and CDR group. Axes are standardized for the same sleep parameter (each column). Participant data separated by low CSF p-tau/Aβ42 (blue) and high CSF p-tau/Aβ42 (red). scEEG vs. actigraphy comparisons are shown for TST, SE, SOL, and WASO (A-D), scEEG vs. diary comparisons are shown for TST, SE, SOL, and WASO (E-H), and actigraphy vs. diary comparisons are shown for TST, SE, SOL, and WASO (I-L).


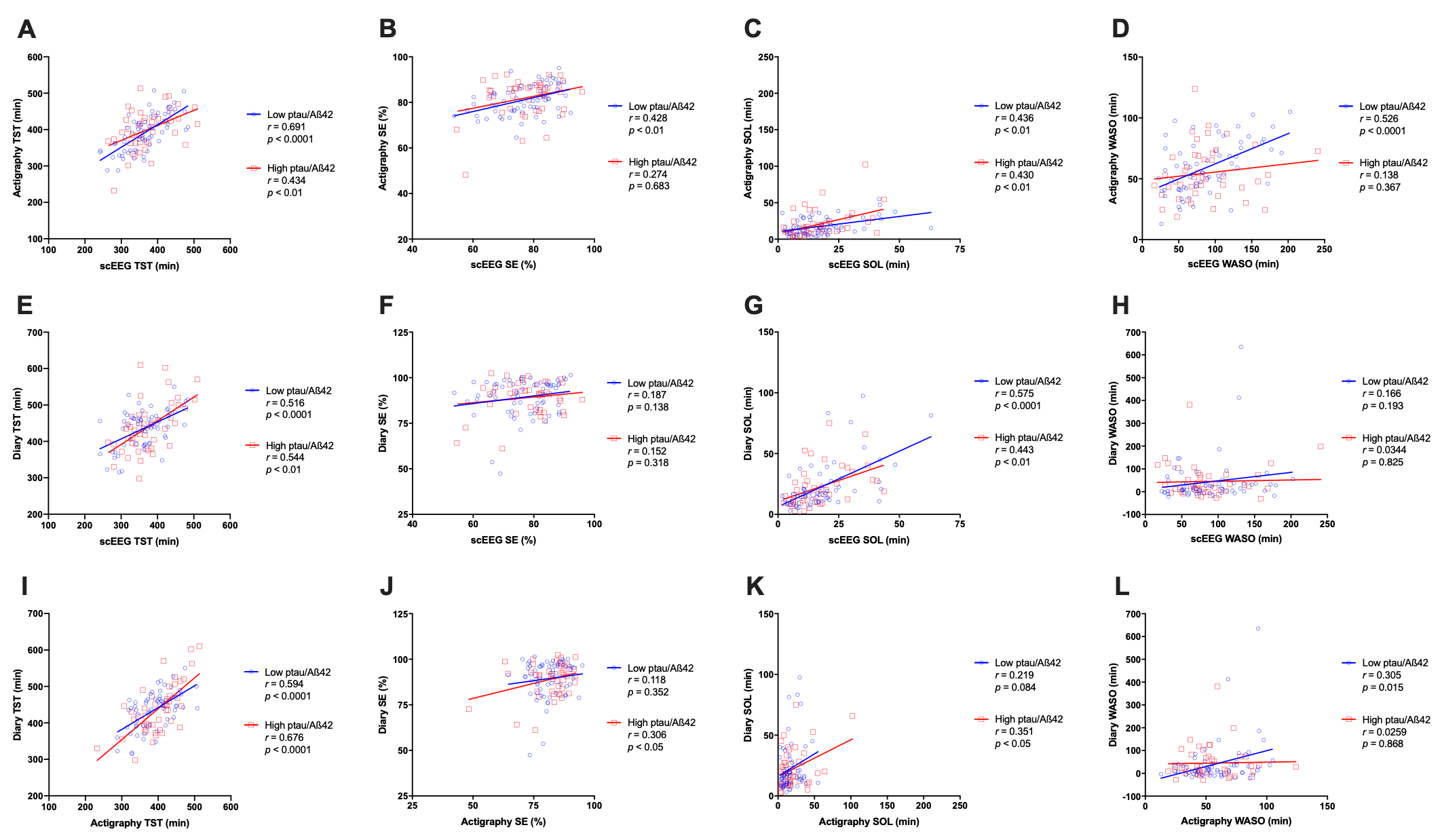


**Supplementary Figure 7. Bland-Altman plots for Total Sleep Time (TST) in the Clinical Dementia Rating (CDR) groups.** Each graph shows the comparison between the average (x-axis) and difference (y-axis) of TST measured by two instruments. The middle dotted line represents the mean bias between the instruments. The upper and lower dotted lines denote the 95% limits of agreement. Each row represents a CDR group in comparing single-channel EEG (scEEG) vs. actigraphy, scEEG vs. diary, and actigraphy vs. diary. A-C: TST for CDR 0. D-F: TST for CDR 0.5.


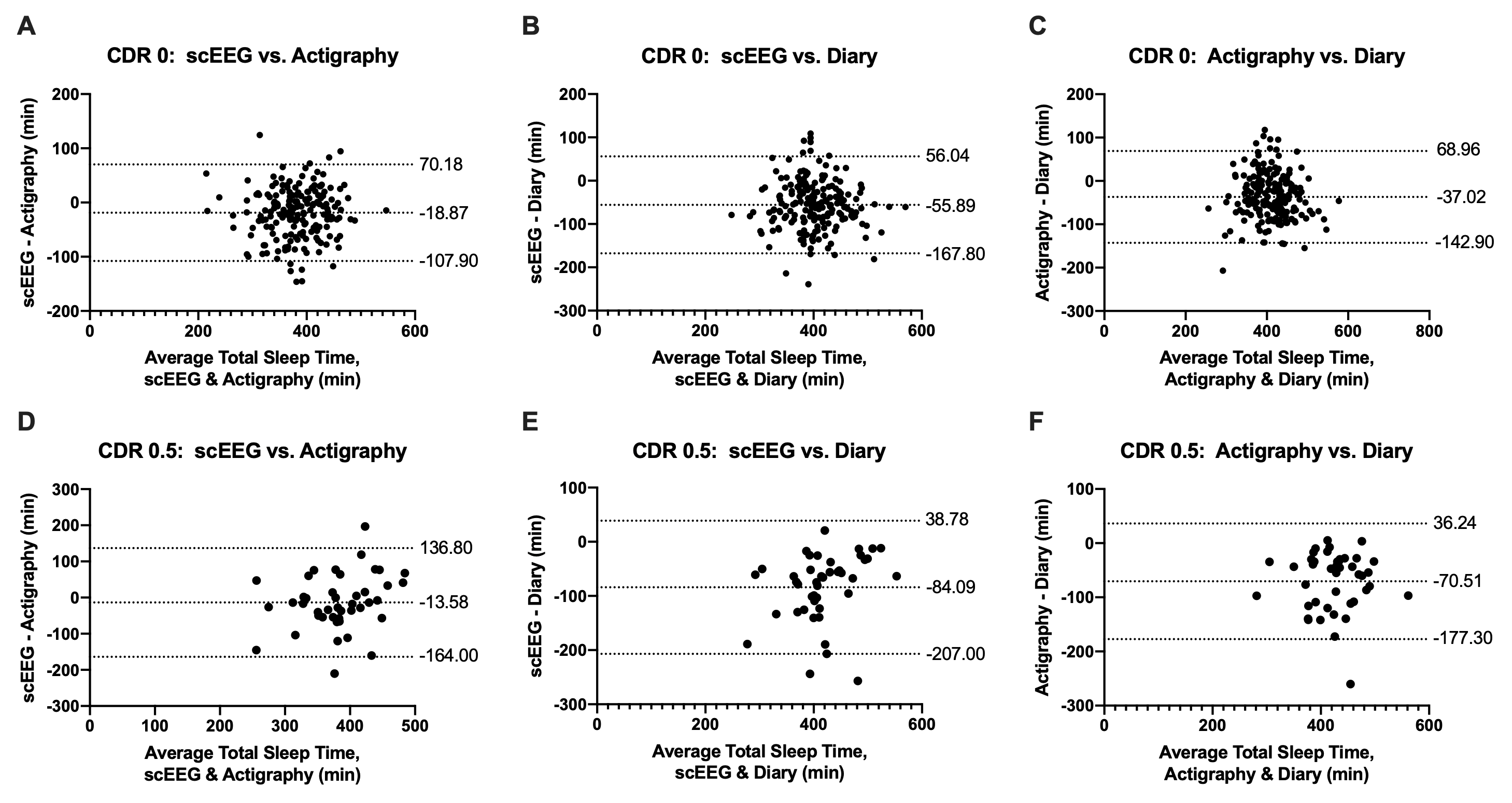


**Supplementary Figure 8. Bland-Altman plots for Sleep Efficiency (SE) in the Clinical Dementia Rating (CDR) groups.** Each graph shows the comparison between the average (x-axis) and difference (y-axis) of SE measured by two instruments. The middle dotted line represents the mean bias between the instruments. The upper and lower dotted lines denote the 95% limits of agreement. Each row represents a CDR group in comparing single-channel EEG (scEEG) vs. actigraphy, scEEG vs. diary, and actigraphy vs. diary. A-C: SE for CDR 0. D-F: SE for CDR 0.5.


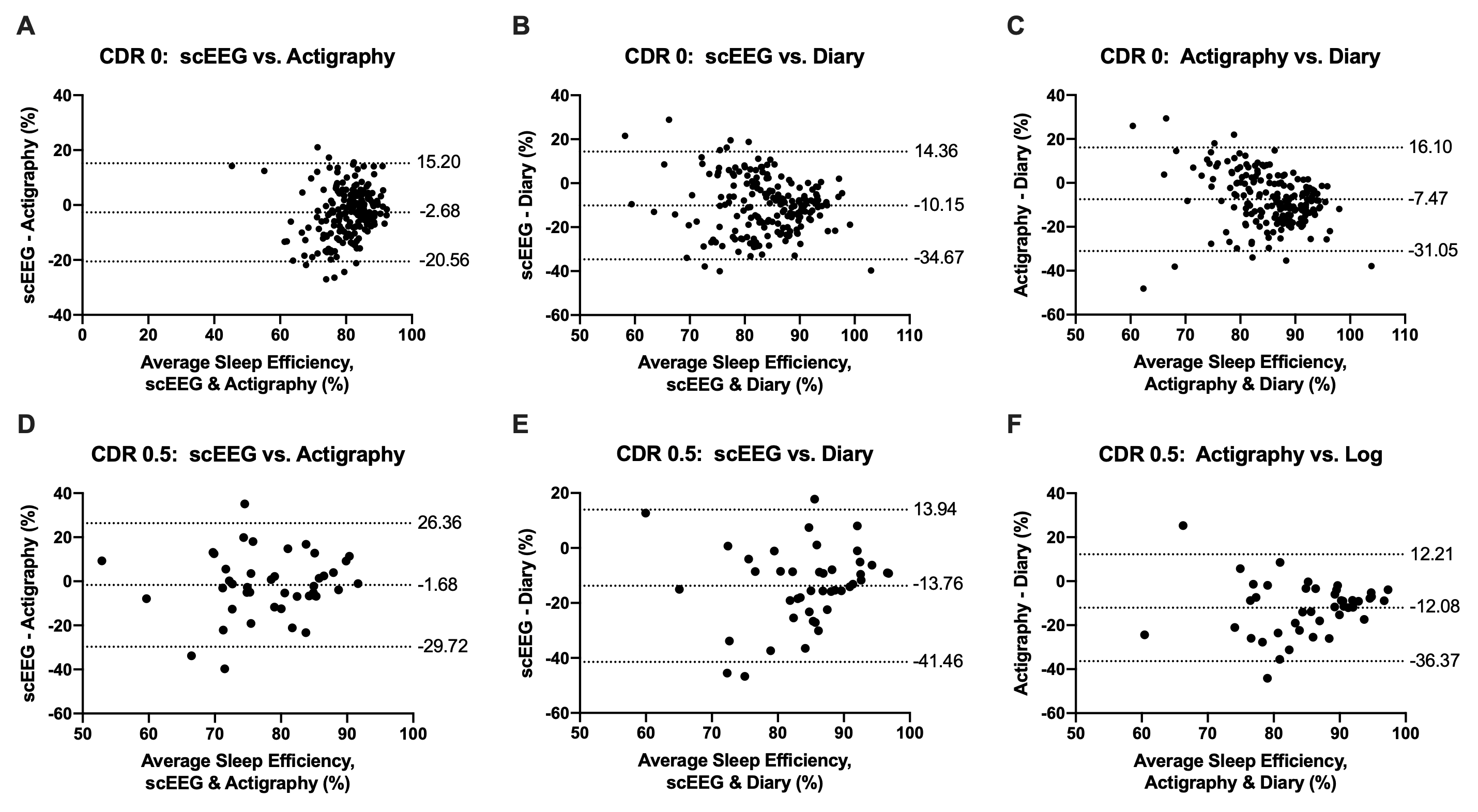


**Supplementary Figure 9. Bland-Altman plots for Sleep Onset Latency (SOL) in the Clinical Dementia Rating (CDR) groups.** Each graph shows the comparison between the average (x-axis) and difference (y-axis) of SOL measured by two instruments. The middle dotted line represents the mean bias between the instruments. The upper and lower dotted lines denote the 95% limits of agreement. Each row represents a CDR group in comparing single-channel EEG (scEEG) vs. actigraphy, scEEG vs. diary, and actigraphy vs. diary. A-C: SOL for CDR 0. D-F: SOL for CDR 0.5.


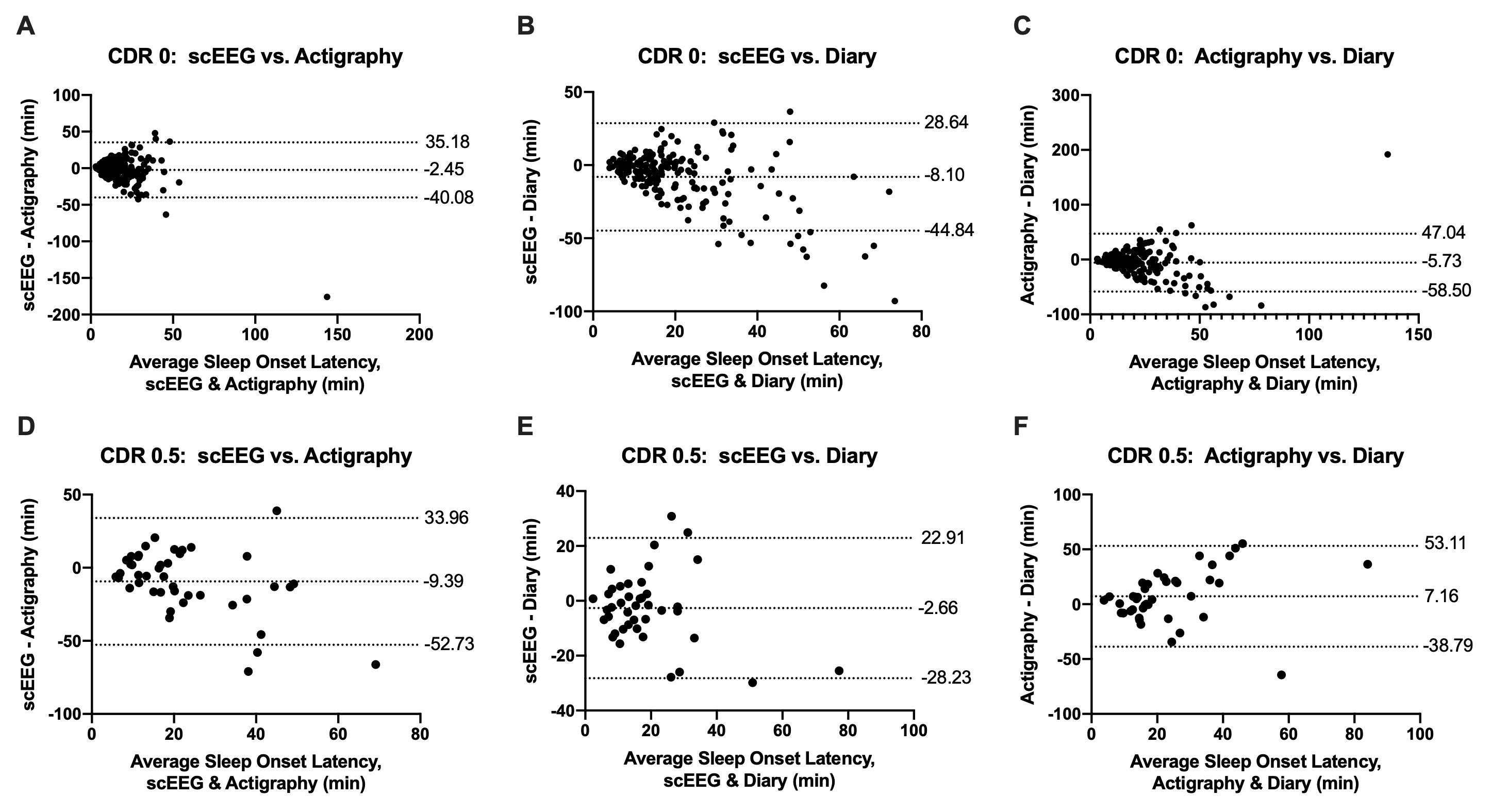


**Supplementary Figure 10. Bland-Altman plots for Wake After Sleep Onset (WASO) in the Clinical Dementia Rating (CDR) groups.** Each graph shows the comparison between the average (x-axis) and difference (y-axis) of WASO measured by two instruments. The middle dotted line represents the mean bias between the instruments. The upper and lower dotted lines denote the 95% limits of agreement. Each row represents a CDR group in comparing single-channel EEG (scEEG) vs. actigraphy, scEEG vs. diary, and actigraphy vs. diary. A-C: WASO for CDR 0. D-F: WASO for CDR 0.5.


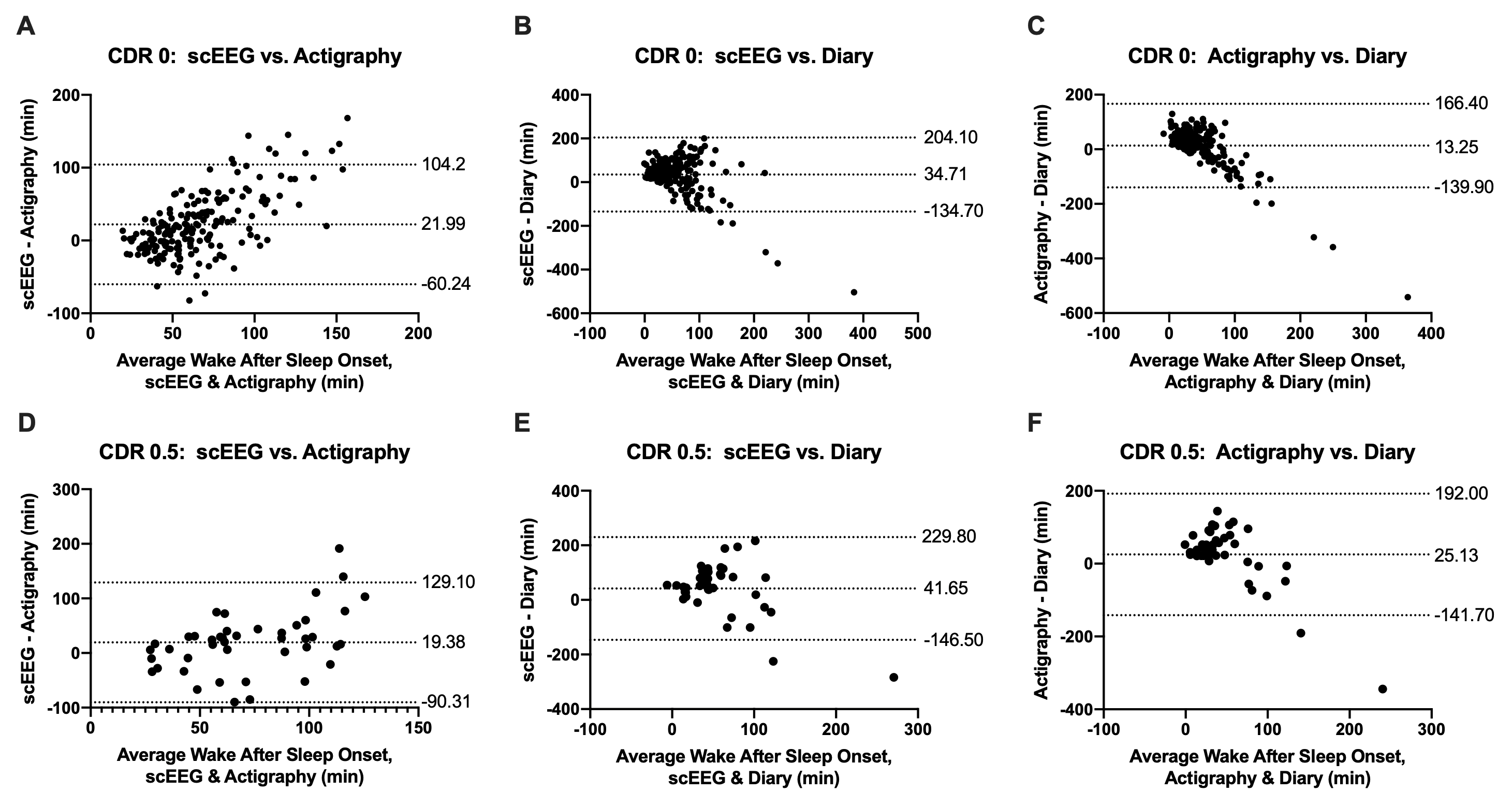


**Supplementary Figure 11. Bland-Altman plots for Total Sleep Time (TST) in the Mini-Mental Status Examination (MMSE) groups.** Each graph shows the comparison between the average (x-axis) and difference (y-axis) of TST measured by two instruments. The middle dotted line represents the mean bias between the instruments. The upper and lower dotted lines denote the 95% limits of agreement. Each row represents a MMSE group in comparing single-channel EEG (scEEG) vs. actigraphy, scEEG vs. diary, and actigraphy vs. diary. A-C: TST for MMSE ≥27. D-F: TST for MMSE <27.


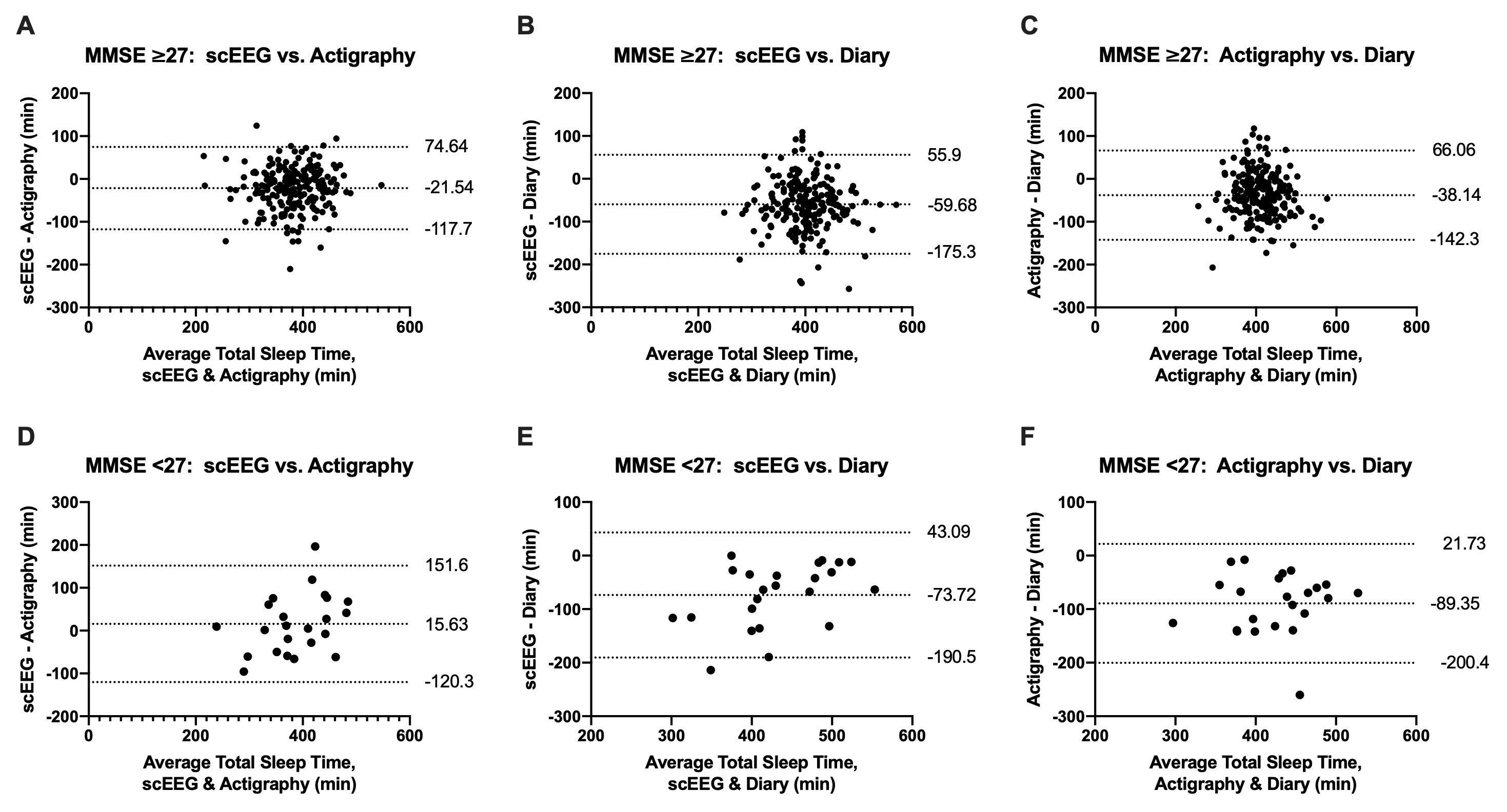


**Supplementary Figure 12. Bland-Altman plots for Sleep Efficiency (SE) in the Mini-Mental Status Examination (MMSE) groups.** Each graph shows the comparison between the average (x-axis) and difference (y-axis) of SE measured by two instruments. The middle dotted line represents the mean bias between the instruments. The upper and lower dotted lines denote the 95% limits of agreement. Each row represents a MMSE group in comparing single-channel EEG (scEEG) vs. actigraphy, scEEG vs. diary, and actigraphy vs. diary. A-C: SE for MMSE ≥27. D-F: SE for MMSE <27.


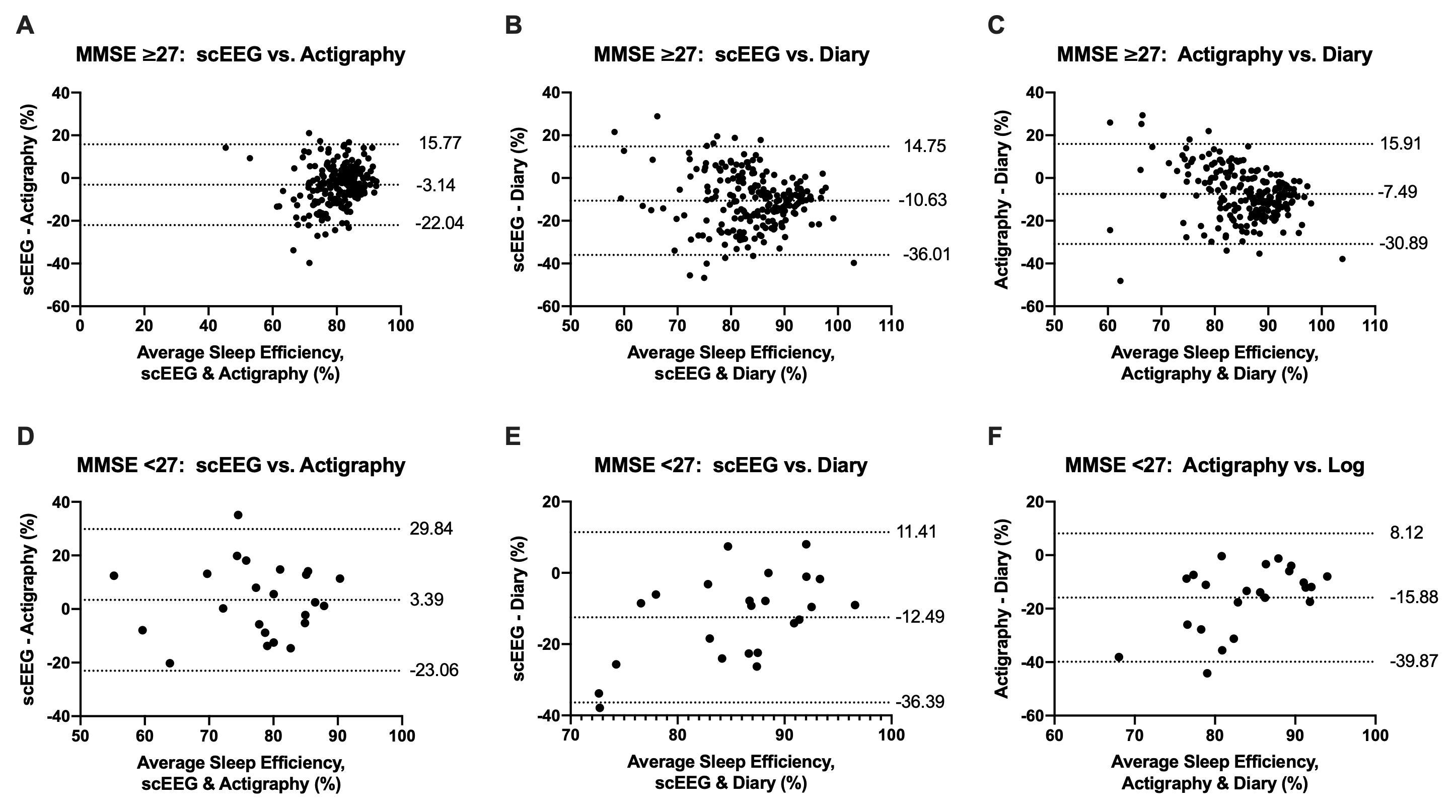


**Supplementary Figure 13. Bland-Altman plots for Sleep Onset Latency (SOL) in the Mini-Mental Status Examination (MMSE) groups.** Each graph shows the comparison between the average (x-axis) and difference (y-axis) of SOL measured by two instruments. The middle dotted line represents the mean bias between the instruments. The upper and lower dotted lines denote the 95% limits of agreement. Each row represents a MMSE group in comparing single-channel EEG (scEEG) vs. actigraphy, scEEG vs. diary, and actigraphy vs. diary. A-C: SOL for MMSE ≥27. D-F: SOL for MMSE <27.


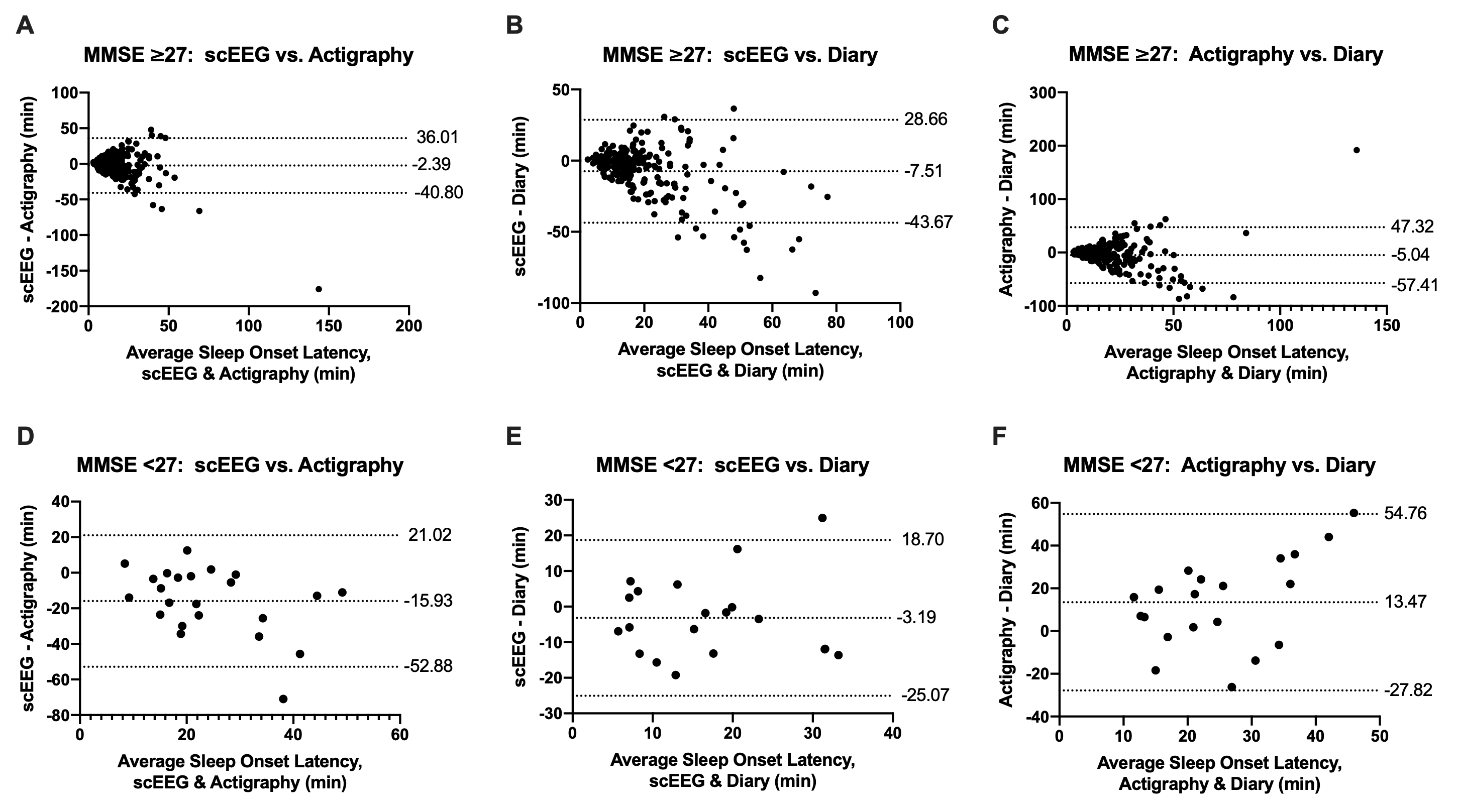


**Supplementary Figure 14. Bland-Altman plots for Wake After Sleep Onset (WASO) in the Mini-Mental Status Examination (MMSE) groups.** Each graph shows the comparison between the average (x-axis) and difference (y-axis) of WASO measured by two instruments. The middle dotted line represents the mean bias between the instruments. The upper and lower dotted lines denote the 95% limits of agreement. Each row represents a MMSE group in comparing single-channel EEG (scEEG) vs. actigraphy, scEEG vs. diary, and actigraphy vs. diary. A-C: WASO for MMSE ≥27. D-F: WASO for MMSE <27.


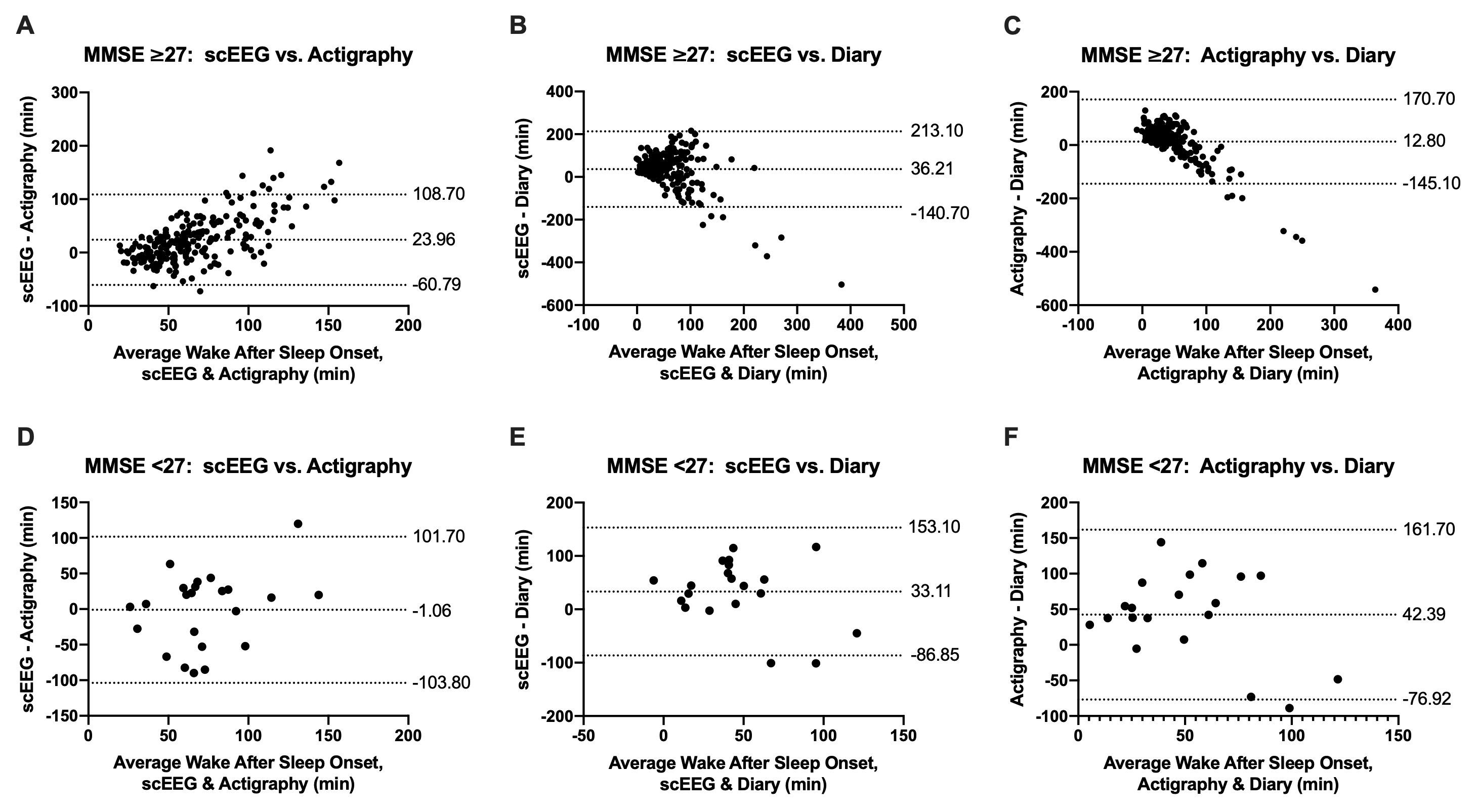


**Supplementary Figure 15. Bland-Altman plots for Total Sleep Time (TST) in the phosphorylated tau (p-tau)/amyloid-β-42 peptide (Aβ42) groups.** Each graph shows the comparison between the average (x-axis) and difference (y-axis) of TST measured by two instruments. The middle dotted line represents the mean bias between the instruments. The upper and lower dotted lines denote the 95% limits of agreement. Each row represents a p-tau/Aβ42 group in comparing single-channel EEG (scEEG) and actigraphy, scEEG and diary, and actigraphy and diary. Axes are standardized across rows. A-C: TST for low p-tau/Aβ42. D-F: TST for high p-tau/Aβ42.


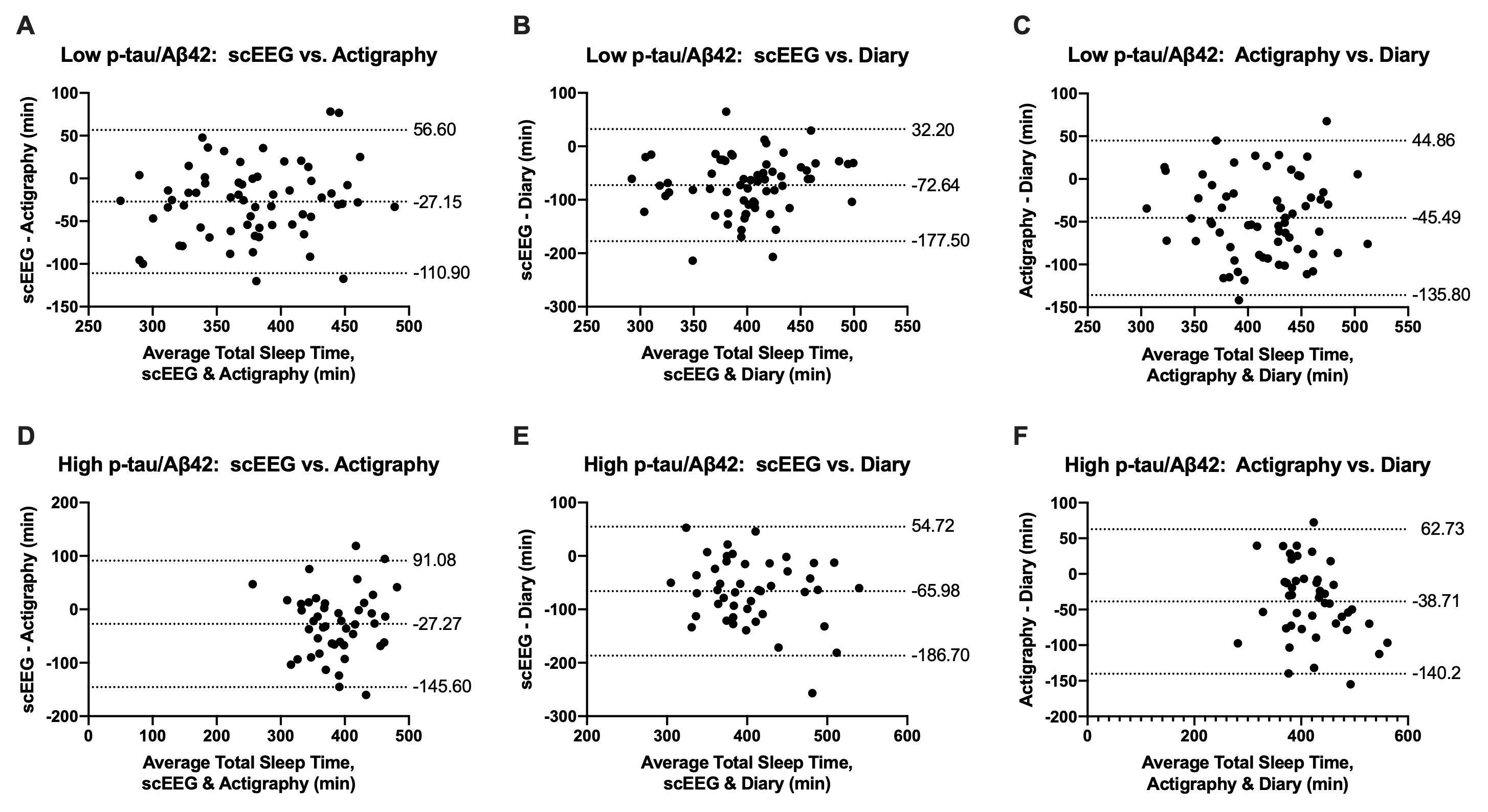


**Supplementary Figure 16. Bland-Altman plots for Sleep Efficiency (SE) in the phosphorylated tau (p-tau)/amyloid-β-42 peptide (Aβ42) groups.** Each graph shows the comparison between the average (x-axis) and difference (y-axis) of SE measured by two instruments. The middle dotted line represents the mean bias between the instruments. The upper and lower dotted lines denote the 95% limits of agreement. Each row represents a p-tau/Aβ42 group in comparing single-channel EEG (scEEG) and actigraphy, scEEG and diary, and actigraphy and diary. Axes are standardized across rows. A-C: SE for low p-tau/Aβ42. D-F: SE for high p-tau/Aβ42.


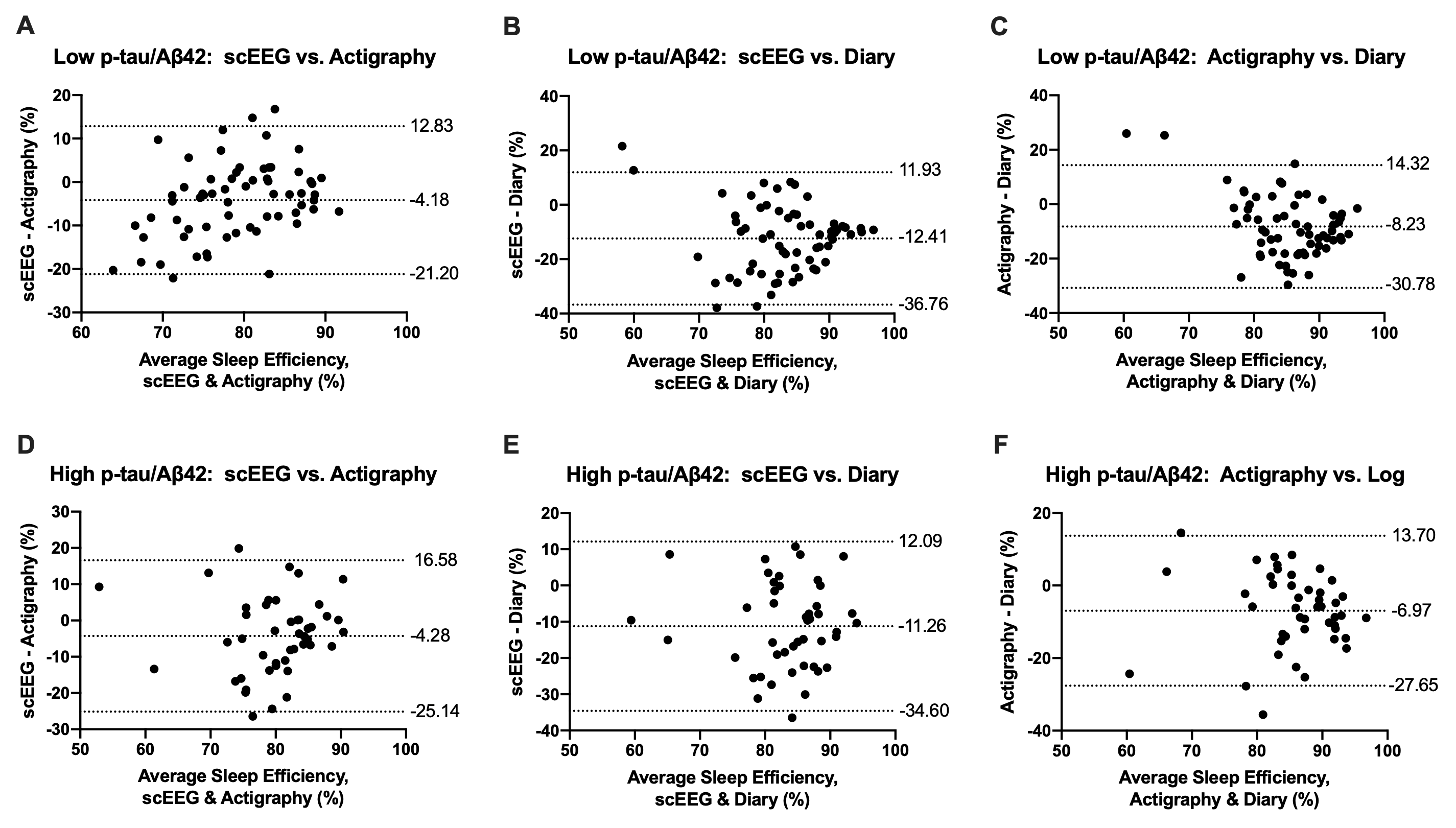


**Supplementary Figure 17. Bland-Altman plots for Sleep Onset Latency (SOL) in the phosphorylated tau (p-tau)/amyloid-β-42 peptide (Aβ42) groups.** Each graph shows the comparison between the average (x-axis) and difference (y-axis) of SOL measured by two instruments. The middle dotted line represents the mean bias between the instruments. The upper and lower dotted lines denote the 95% limits of agreement. Each row represents a p-tau/Aβ42 group in comparing single-channel EEG (scEEG) and actigraphy, scEEG and diary, and actigraphy and diary. Axes are standardized across rows. A-C: SOL for low p-tau/Aβ42. D-F: SOL for high p-tau/Aβ42.


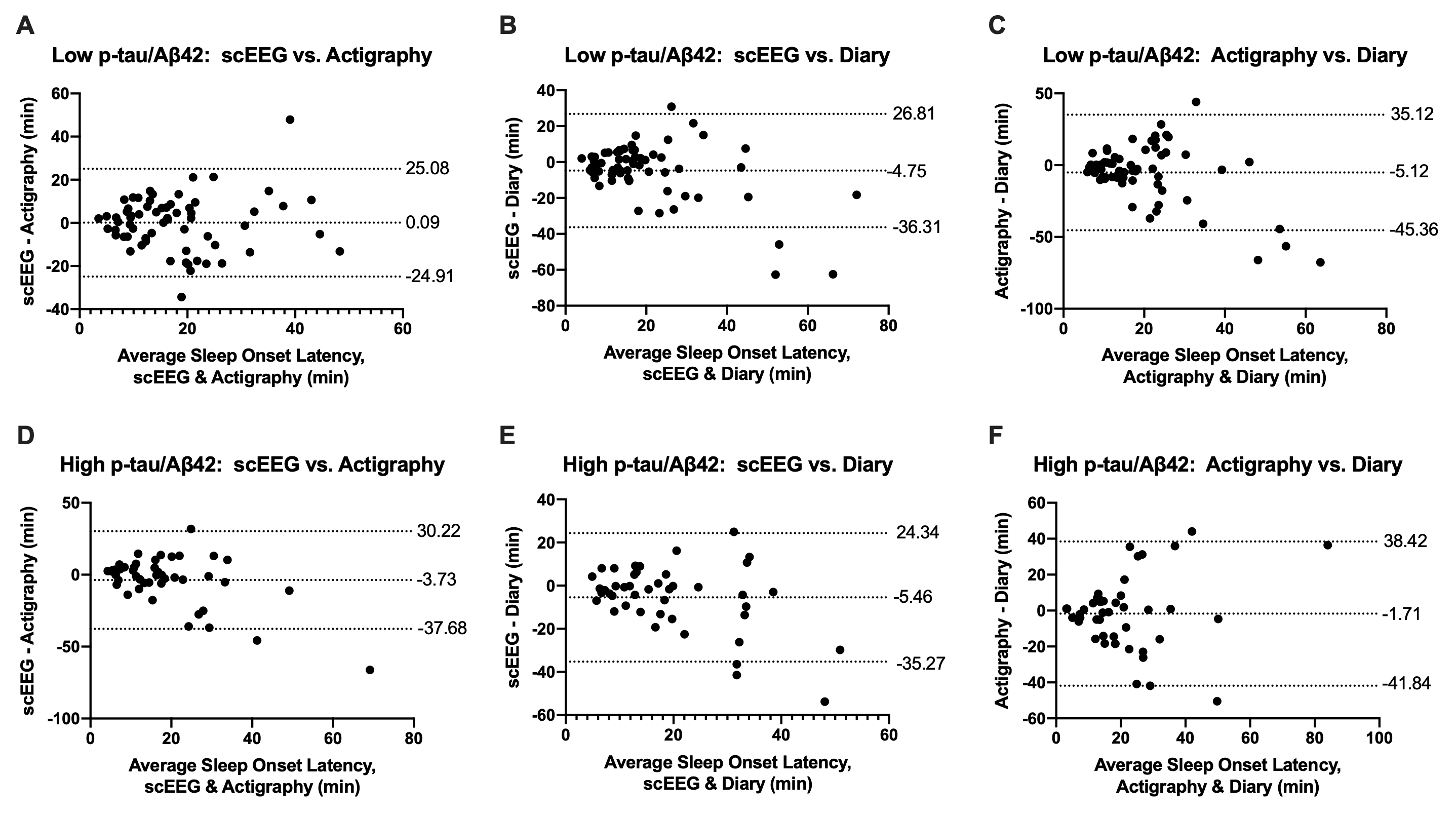


**Supplementary Figure 18. Bland-Altman plots for Wake After Sleep Onset (WASO) in the phosphorylated tau (p-tau)/amyloid-β-42 peptide (Aβ42) groups.** Each graph shows the comparison between the average (x-axis) and difference (y-axis) of WASO measured by two instruments. The middle dotted line represents the mean bias between the instruments. The upper and lower dotted lines denote the 95% limits of agreement. Each row represents a p-tau/Aβ42 group in comparing single-channel EEG (scEEG) and actigraphy, scEEG and diary, and actigraphy and diary. Axes are standardized across rows. A-C: WASO for low p-tau/Aβ42. D-F: WASO for high p-tau/Aβ42.


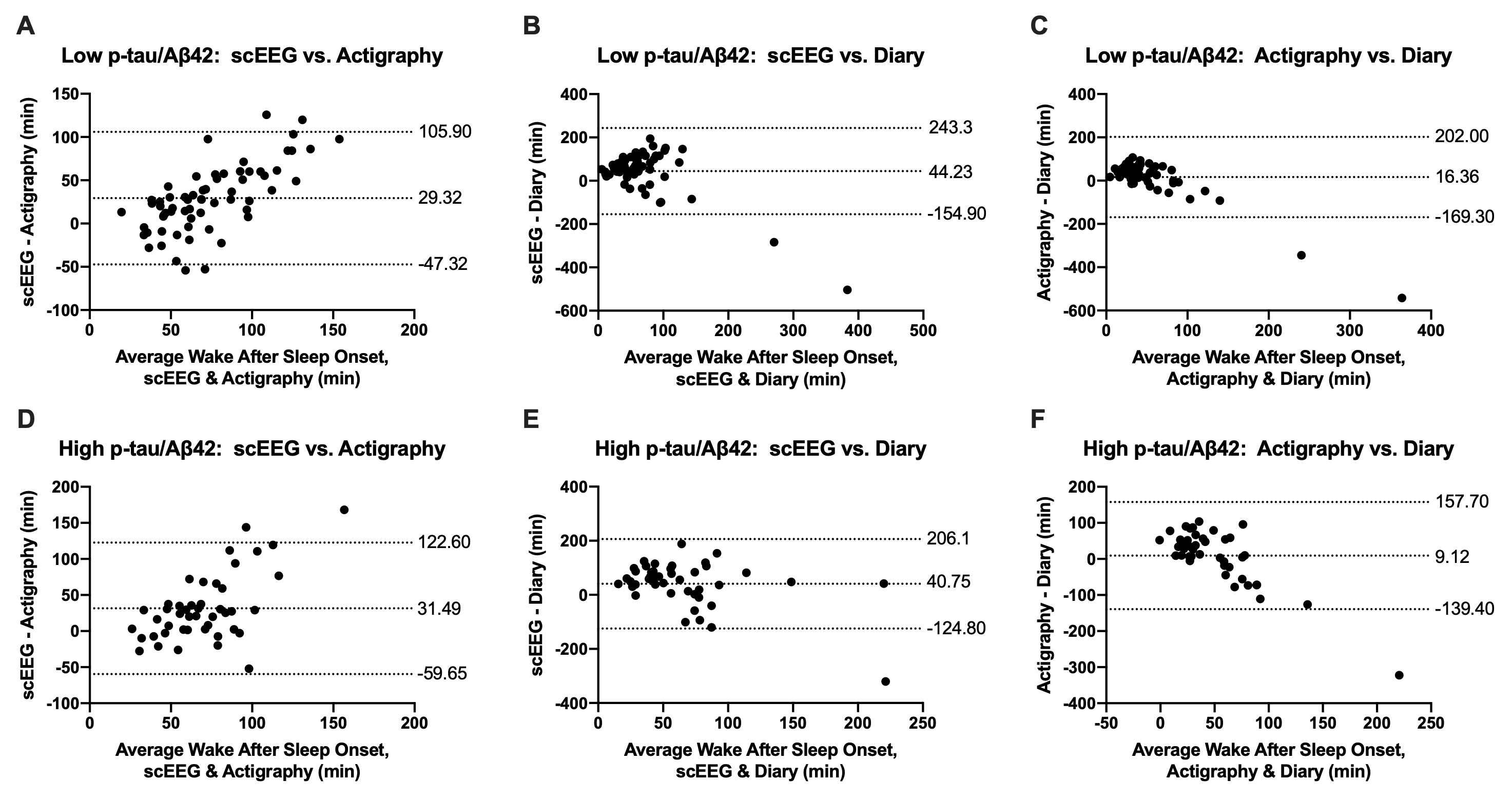

Supplement: zpaa006_suppl_Supplementary_Materials [file zpaa006_suppl_supplementary_materials.docx]
